# Metagenomic editing of commensal bacteria *in vivo* using CRISPR-associated transposases

**DOI:** 10.1126/science.adx7604

**Published:** 2025-11-13

**Authors:** Diego Rivera Gelsinger, Carlotta Ronda, Junjie Ma, Om B. Kar, Madeline Edwards, Yiming Huang, Chrystal Mavros, Yiwie Sun, Tyler Perdue, Phuc Leo Vo, Ivaylo I. Ivanov, Samuel H. Sternberg, Harris H. Wang

**Affiliations:** 1 Department of Systems Biology, Columbia University, New York, NY, USA; 2 Department of Biochemistry and Molecular Biophysics, Columbia University, New York, NY, USA; 3 Department of Microbiology and Immunology, Vagelos College of Physicians and Surgeons, Columbia University, New York, NY 10032, USA; 4 Department of Biological Sciences, Columbia University, New York, NY, USA; 5 Department of Molecular Pharmacology and Therapeutics, Columbia University, New York, NY, USA; 6 Howard Hughes Medical Institute, Columbia University, New York, NY, USA; 7 Department of Pathology and Cell Biology, Columbia University, New York, NY, USA; 8 Present address: Innovative Genomics Institute, University of California Berkeley, Berkeley, CA, USA; 9 Present address: Foli Bio Inc., New York, NY, USA; 10 Present address: Vertex Pharmaceuticals, Inc, Boston, MA, USA

## Abstract

Metagenomic sequencing has revealed a rich microbial biodiversity within the mammalian gut. Current approaches to genetically alter the gut microbiome to produce new functional changes are highly limited, owing to a lack of molecular tools that differentiate one microbe from another. Here, we introduce ‘metagenomic editing’ (MetaEdit) as a platform technology for microbiome engineering. This approach employs optimized CRISPR-associated transposases delivered by a broadly conjugative vector to modify targeted native Gram-negative and Gram-positive gut bacteria with large site-specific integrated payloads, including entire new pathways. With MetaEdit, we demonstrate the *in vivo* ‘genetic capture’ of a native murine *Bacteroides* using a metabolic payload that enables new functional growth control with dietary inulin. Furthermore, we show *in vivo* genomic modification of Segmented Filamentous Bacteria (SFB), a mucosa-associated and potent immunomodulatory bacterium recalcitrant to laboratory cultivation. MetaEdit endowed SFB with GFP fluorescence to enable identification and enrichment of the engineered strain. Collectively, this work provides a paradigm to precisely manipulate the genome of individual bacteria in native communities that contain gigabases of metagenomic repertoire.

## One-Sentence Summary

Metagenomic editing with mobile CRISPR-associated transposases enables precise and durable genetic engineering of targeted bacteria in the gut of live animals.

## INTRODUCTION

The gut microbiome is composed of hundreds to thousands of unique bacterial species that, in aggregate, rival the human genome in size and supply a dynamic secondary genetic reservoir for the host (1, 2). These microbes possess diverse metabolic and functional capabilities that influence dietary digestion, drug interactions, immunity, and disease susceptibility, yet many of these processes remain poorly understood (3–6). Unlocking the microbiome’s genetic potential could reveal fundamental host-microbe mechanisms and enable the development of targeted therapeutic interventions (2, 7–9).

Current strategies to manipulate the gut microbiome have major limitations (10, 11). Antibiotics and fecal microbiota transplantation (FMT) broadly reshape microbial composition but lack specificity and can have unintended effects (12, 13). More targeted approaches, such as bacteriophage therapy and CRISPR-Cas9-based gene editing, can make small-scale edits or induce cell killing, but cannot produce pathway-scale changes (14–16). Engrafting desired biological functions directly into natively abundant microbes (17) is an approach that has yet to be fully explored, particularly with large multi-gene operons that are several kilobases in size as payloads.

The recent discovery of CRISPR-associated transposases (CAST) has enabled programmable integration of multi-kilobase DNA payloads into defined genomic loci (18–20). Building upon these breakthroughs, we present here **Meta**genomic **Edit**ing (**MetaEdit**), a powerful approach that leverages CAST to directly engineer specific bacteria in the native microbiome *in vivo*. We demonstrate the ability to edit human and murine gut bacteria, control their gut colonization capacity, and modify a mucosa-associated, immunomodulatory bacterium, Segmented Filamentous Bacteria (SFB). MetaEdit is a foundational technology that can accelerate new fundamental microbiome research and support microbiome-based medicines.

## RESULTS

### Development and optimization of MetaEdit

**Fig. 1.**
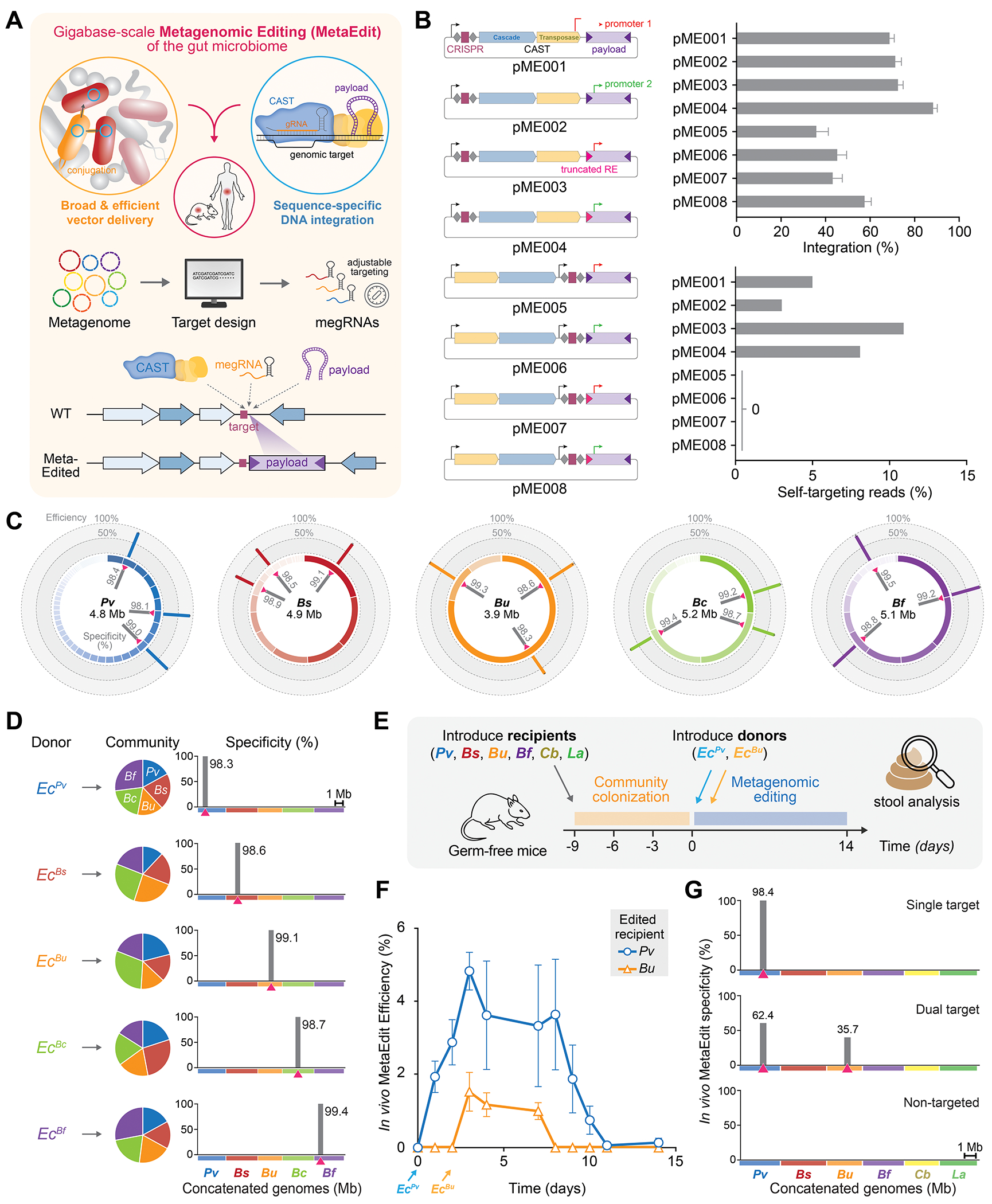
Development and optimization of MetaEdit for gut microbiome engineering. (**A**) Schematic of MetaEdit where CAST machinery and megRNA coordinate the integration of a payload into target bacteria within a microbiome. (**B**) Diagram of eight pME vector designs with varying promoter sequence, transposon ends, CAST gene position and their corresponding integration efficiency and self-targeting frequency tested in *B. vulgatus*. (**C**) Circos genome plots for five human *Bacteroidaceae* isolates: *P. vulgatus* (*Pv*), *B. stercoris* (*Bs*), *B. uniformis* (*Bu*), *B. caccae* (*Bc*), and *B. fragilis* (*Bf*). Discrete genomic contig lengths are plotted as the inner ring. Three megRNA target sites (pink triangles) and their integration specificity (inner bar) and efficiency (outer bar) are shown. (**D**) Characterization of MetaEdit integration into a synthetic community of five *Bacteroidaceae* using distinct *E. coli* donors (*Ec*^*recipient*^). Community compositions are shown as pie charts (left) and integration specificities across the concatenated recipient genomes are shown as bar plots (right) with intended target sites (pink triangles). (**E**) Experimental workflow for MetaEdit in gnotobiotic mice colonized with a 6-member human gut community. (**F**) Resulting MetaEdit integration efficiency in *Pv* and *Bu* target strains in the gut treated with donor *Ec*^*Pv*^ at Day 0 and *Ec*^*Bu*^ at Day 2 as measured by qPCR from fecal matter. (**G**) Day 3 integration specificities (%) of single *Pv* target cohort or dual *Pv*/*Bu* targets cohort compared to non-target (NT) control cohort across the community. Integration efficiency refers to the amount of detected wild-type that has been edited. Integration specificity refers to the amount of payload that is found at the on-target site compared to the rest of the (meta)genome. Integration efficiency data in (B–G) are shown as mean ± s.d. for n=3 independent biological or mouse replicates and deep sequencing specificity data are shown by a representative replicate.

Metagenomic editing of the complex gut microbiome requires the synthesis of several key properties: delivery, precision, persistence, and host compatibility (Fig. 1A, Fig. S1). The genetic payload must first be efficiently delivered to a target strain while withstanding the harsh gut environment, interference from other bacteria, enzymatic and chemical degradation, immune responses, and washout due to intestinal motility. We recently described an efficient delivery approach using bacterial conjugation via an IncPα-family RP4 system, enabling the transfer of genetic payloads to both Gram-negative and Gram-positive microbes in the mammalian gut (21). We therefore adopted a similar strategy in which a MetaEdit plasmid (pME) carries the payload and is mobilized by an engineered donor *E. coli* EcGT2 containing the RP4 conjugation machinery. EcGT2 is genomically tagged with a mCherry gene for tracking and modified with diaminopimelic acid (DAP) auxotrophy to restrict its replication in the gut while facilitating *in vitro* selection and fluorescence-based analysis (21).

For long-term retention and avoiding collateral damage to other microbiota, target specificity is crucial for genomic editing since off-target integrations lead to fitness defects and negative selection, often observed with conventional transposases that can randomly disrupt essential genes (22). The delivered payload must also integrate precisely into the recipient genome before pME is lost through successive cell divisions. We employed CAST, a versatile and programmable RNA-guided DNA integration system, to insert the payload from pME into the recipient genome via a cut-and-paste transposition mechanism. Importantly, if the designated insertion site is absent in the recipient’s genome, the payload is not integrated and is rapidly lost along with pME. This precise genomic integration is essential for long-term payload persistence. Finally, the payload must function within the target bacterium using compatible regulatory and genetic elements to add another layer of specificity and orthogonality to metagenomic editing.

To establish CAST activity in gut bacteria, we used species from the *Bacteroidaceae* family since they are dominant members of the gut microbiome. We first optimized replicative pME vectors in *Phocaeicola vulgatus* (*Pv*) by exploring key genetic elements that maximized payload integration efficiency, including the promoter sequence, transposon ends, and the position of CAST genes relative to the CRISPR array (Fig. S2, Methods). Self-targeting insertion is a phenomenon we previously reported where CAST targets the spacer found within its CRISPR array, leading to low-level off-target insertions that inactivate the system (23, 24). Vectors with CRISPR arrays positioned upstream (pME0001-0004) exhibited the highest integration efficiency (>70%) but also induced self-targeting insertions (Fig. 1B, S2A–B). In contrast, designs placing the transposon ends adjacent to the CRISPR arrays (pME0005-0008) reduced integration efficiency but eliminated self-targeting (Fig. 1B, S2A–B). The optimal configuration (pME0008) achieved >50% integration efficiency while maintaining minimal self-targeting (Fig. 1B).

To tune editing specificity, we developed a computational pipeline for designing MetaEdit gRNAs (megRNAs), in which 32-nt spacer candidates are identified by mapping *k-mers*, potential off-targets designs are eliminated, and on-target specificity is maximized using whole genomes or metagenomically assembled genomes (MAGs) (Fig. S3, Methods). With this approach, we designed and tested three megRNAs for each of five human gut *Bacteroidaceae* isolates: *P. vulgatus* (*Pv*), *B. stercoris* (*Bs*), *B. uniformis* (*Bu*), *B. caccae* (*Bc*), and *B. fragilis* (*Bf*). CAST-mediated payload integration with these megRNAs demonstrated nearly 100% specificity and high efficiency at the intended genomic site, as confirmed by deep sequencing with a modified Tn-seq protocol and qPCR measurements (Fig. 1C, Methods). To further validate strain-level specificity and orthogonality, we assembled a synthetic community comprising all five *Bacteroidaceae* species and separately introduced donor strains carrying a megRNA-targeted payload against one specific strain (Fig. 1D). Bioinformatic analysis of integration sites across this synthetic community confirmed that each megRNA exclusively targeted its intended site and strain, demonstrating the ability to perform precise, taxa-selective editing within a multi-species microbial consortium (Fig. 1D).

We next sought to test MetaEdit *in vivo* in a complex defined gut community using a mouse model. Gnotobiotic mice were first colonized with four Gram-negative *Bacteroidaceae* strains and two Gram-positive bacteria, *Clostridia bolteae* (*Cb*) and *Lactobacillus animalis* (*La*) (Fig. 1E, Fig. S4). Two donors strains, *Ec^Pv^* and *Ec^Bu^*, carrying megRNAs targeting *Pv* or *Bu,* respectively, were introduced sequentially at 10^10^ cells per mouse on Day 0 and Day 2. MetaEdit efficiency and specificity were assessed using qPCR and sequencing using fecal samples as a proxy for *in vivo* conditions. To improve the detection of payload integration events in fecal samples, we optimized a qPCR protocol that yielded an integration sensitivity as low as 0.1% (Fig. S5A–B). Additionally, we developed an enhanced Tn-seq method (TagTn-seq) for metagenome-wide integration detection, using tagmentation and nested amplicons to improve sensitivity down to 0.01% (Fig. S5C–E). With these improvements, we observed payload integration in the gut peaking at ~5% within 3 days for the *Pv*-targeted construct, and payload persistence for up to 10 days (Fig. 1F). Notably, 98.4% of all integrations were on-target events to the intended *Pv* genome insertion site (Fig. 1G). When the second donor (*Ec^Bu^*) was introduced on Day 2, we observed a subsequent increase in *Bu*-specific payload integration (~2%) at its designated *Bu* genomic site with no cross-targeting between species (Fig. 1F–G). These results demonstrate that MetaEdit is capable of microbiome gene delivery and insertion into multiple strains in the gut with high efficiency and species-resolution using carefully designed mobile CAST and megRNA components.

### Metagenomic editing of the native gut microbiome

**Fig. 2:**
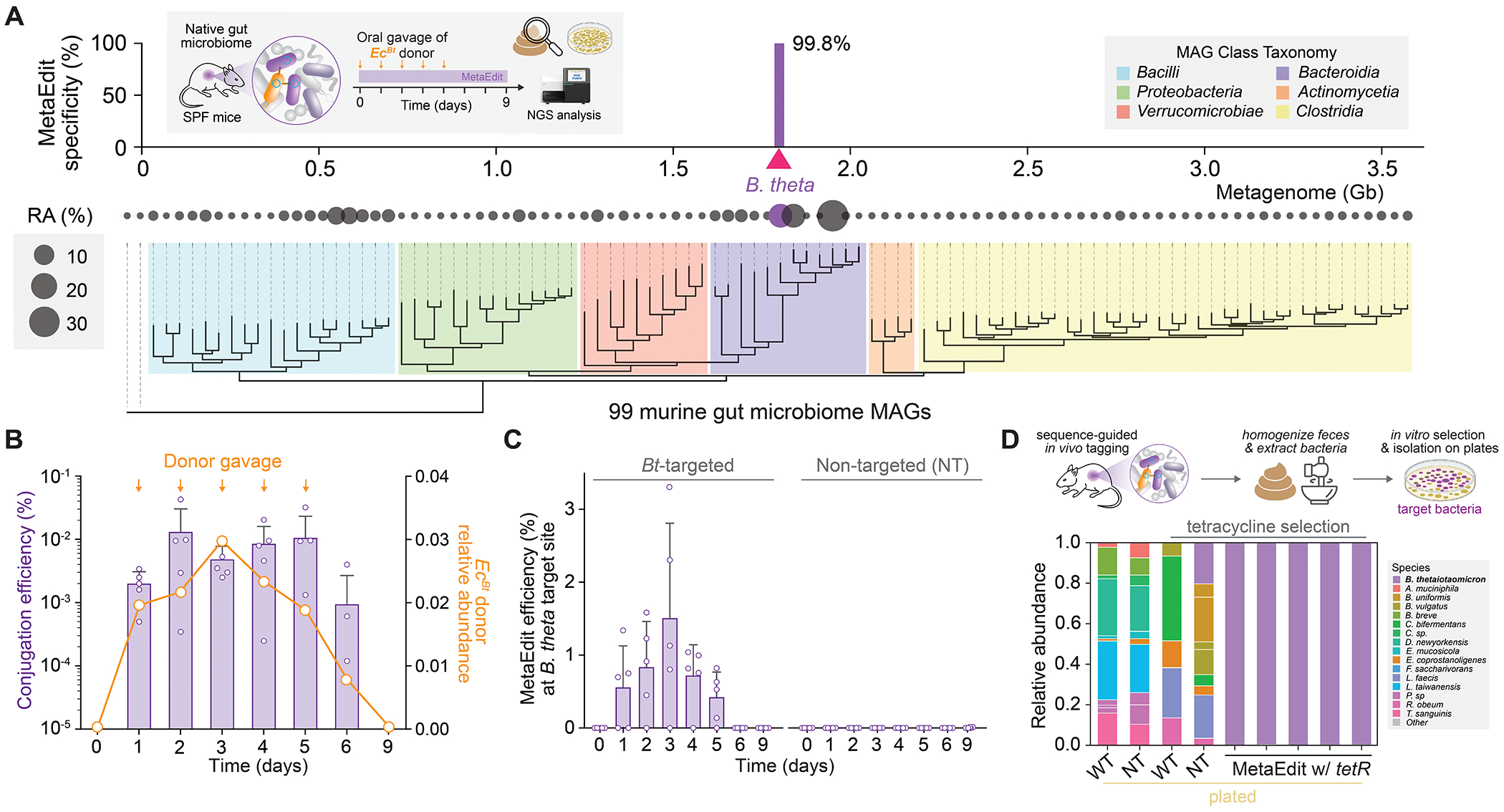
Targeted *in vivo* engineering and tagging of native gut bacteria. (**A**) MetaEdit experiment targeting a native murine microbe (inset). Phylogenetic tree and relative abundance (RA %) of all metagenome-assembled genomes (bottom) from a mouse microbiome overlayed with integration specificity (%) for targeted editing of the native *B. thetaiotaomicron* (top) across the 3.6 Gb concatenated metagenome. (**B**) Conjugation efficiency of MetaEdit vectors (left axis) and relative abundance of donor (right axis) over time within the murine gut. Donor relative abundance was calculated through metagenomics on fecal extracts and conjugation efficiency was calculated by platting fecal samples ± the pME backbone marker. (**C**) Integration efficiencies in *Bt* between mice cohorts treated with a targeted donor (*Ec*^*Bt*^) or a non-targeting megRNA donor control by qPCR over time. (**D**) Schematic for payload-tagging strains within a microbiome for sequence-guided isolation (top), and 16S relative abundance plots on plated colonies isolated from fecal samples ± antibiotic payload selection (bottom). Plated fecal sample conditions are denoted as untreated (WT), non-targeted pME-treated (NT), and *Bt*-targeted pME-treated (MetaEdit w/ *tetR*) mice, respectively. Relative abundance only captures gut bacteria that can grow on plates. Conjugation efficiency and integration efficiency data in (B–C) are shown as mean ± s.d. for n=3 independent mouse replicates and deep sequencing specificity data are shown as replicates.

To assess whether MetaEdit can access and manipulate specific species within a complex microbiome, we next explored targeting a native gut bacterium amongst hundreds of other species across billions of base pairs. Given its prevalence in the Jackson Labs mouse gut (~12% average relative abundance as determined by metagenomic sequencing) and its extensive genetic repertoire for dietary fiber metabolism, *Bacteroides thetaiotaomicron* (*Bt*) was chosen as a target for MetaEdit (Fig. 2A, Fig. S6). Based on the sequenced metagenome of specific pathogen-free (SPF) C57BL/B6 mice, we designed and constructed a vector (pME0024) carrying megRNA that targeted *Bt* at a neutral intergenic region downstream of the *glmS* gene. *glmS* was chosen because CASTs and Tn7-like transposons are commonly found in nature to be encoded downstream of *glmS* or tRNAs (25–27). We hypothesized that through selective pressure, this site likely exhibits a neutral effect on the host allowing for the transposable element to remain within the bacterial host genome. This plasmid was introduced to the EcGT2 strain to produce the *Ec^Bt^* donor, which was orally administered daily to SPF mice at 10^10^ cells for 5 days (Fig. 2A **inset**).

To evaluate conjugation efficiency and donor persistence, we tracked *Ec^Bt^* donor and pME0024-transfer kinetics via selective plating and sequencing over time. Conjugation efficiency *in vivo* ranged from 10^−2^ to 10^−3^, and donor cells were not detected by Day 9 (i.e., 4 days post-final dose) (Fig. 2B), consistent with our prior observations (21). Across the 3.6 billion base pairs of metagenomic sequence of this gut community, we observed 99.8% on-target payload integration into the native *Bt* (Fig. 2A). These highly specific integration events were consistently observed across multiple mice over time (Fig. S7). Average payload integration efficiency peaked at ~1.5% and could be detected for five days, compared to no integration with a non-targeting megRNA control (Fig. 2C). We also designed a megRNA (pME0025) targeting the gene body of a bile salt hydrolase gene (LNGAGAPF_04089) in *Bt* , which showed similar levels of integration specificity, efficiency, and expression of the payload (Fig. S8). These results confirm that MetaEdit directs precise genomic integration and expression of a heterologous payload into native gut bacteria *in vivo*.

We hypothesized that the genetically-tagged *Bt* strain could be selectively isolated from the complex community. Directly plating fecal extracts on non-selective media and sequencing the resulting colonies in bulk did not reveal any native *Bt* (Fig. 2D). Strikingly, plating on tetracycline selective media yielded only MetaEdited *Bt* isolates carrying the *tetR* payload. PCR detection of the integration junction and vector backbone further confirmed that the *Bt* isolates were genetically tagged by MetaEdit compared to non-targeting (NT) control isolates (Fig. S9). These findings demonstrate the selective isolation of a target bacterium using only a unique 32-bp region of its genome from an otherwise highly complex microbiome—a new capability that can expand microbial culturomics efforts (20, 28).

We surmised that edited strains were outcompeted *in vivo* due to a fitness burden imposed by MetaEdit. Potential causes included toxicity from genomic integration, metabolic burden from maintaining the 15–17 kb MetaEdit vector, or constitutive overexpression of the CAST operon and payload. To pinpoint the source, we deconstructed the system into four components: (1) the cured clonal integrant, (2) a pME minimal backbone with the oriT and rep open reading frame (ORF), (3) a payload-only pME, and (4) a CAST-only pME (Fig. S10). Growth profiling revealed that constitutive expression of the CAST operon imposed the strongest fitness cost, with some lesser additional burden from strong overexpression of the payload (Fig. S10). These results suggest that metabolic load from sustained expression is a key driver of negative selection against edited strains.

### Controlling in vivo engineered bacteria with metabolic payloads

A key challenge in microbiome engineering is controlling and maintaining the desired function of an exogenous bacterium in the native community—a hurdle MetaEdit can address through large edits. Our previous *in vivo* efforts relying on conjugative plasmid delivery resulted in transconjugants that vanished within 48 hours (21). With MetaEdit, we can achieve more durable edits in both gnotobiotic and SPF mice over 5-10 days (Fig. 1F, Fig. 2C), but the modified bacteria ultimately disappeared likely due to competition with their wild-type counterparts. Bacteria encode various polysaccharides utilization loci (PUL) that can be exploited to enhance growth and colonization in the gut (29–32). We therefore hypothesized that integrating a PUL payload into native *Bt* could enable precise control of its abundance *in vivo* in response to a dietary supplement and tune the persistence of the engineered function. To explore this concept, we first tested the ability of different SPF murine gut microbiota (Charles River, Jackson Laboratory, Taconic) to grow on common dietary polysaccharides (Fig. S11, Methods). While levan promoted growth in all three communities, xylan elicited moderate growth in only the Taconic microbiome (Fig. S11C). Inulin, a fermentable fiber, failed to promote growth for all microbiota, resembling unsupplemented controls—suggesting its potential as a selective metabolic substrate (Fig. S11).

**Fig. 3:**
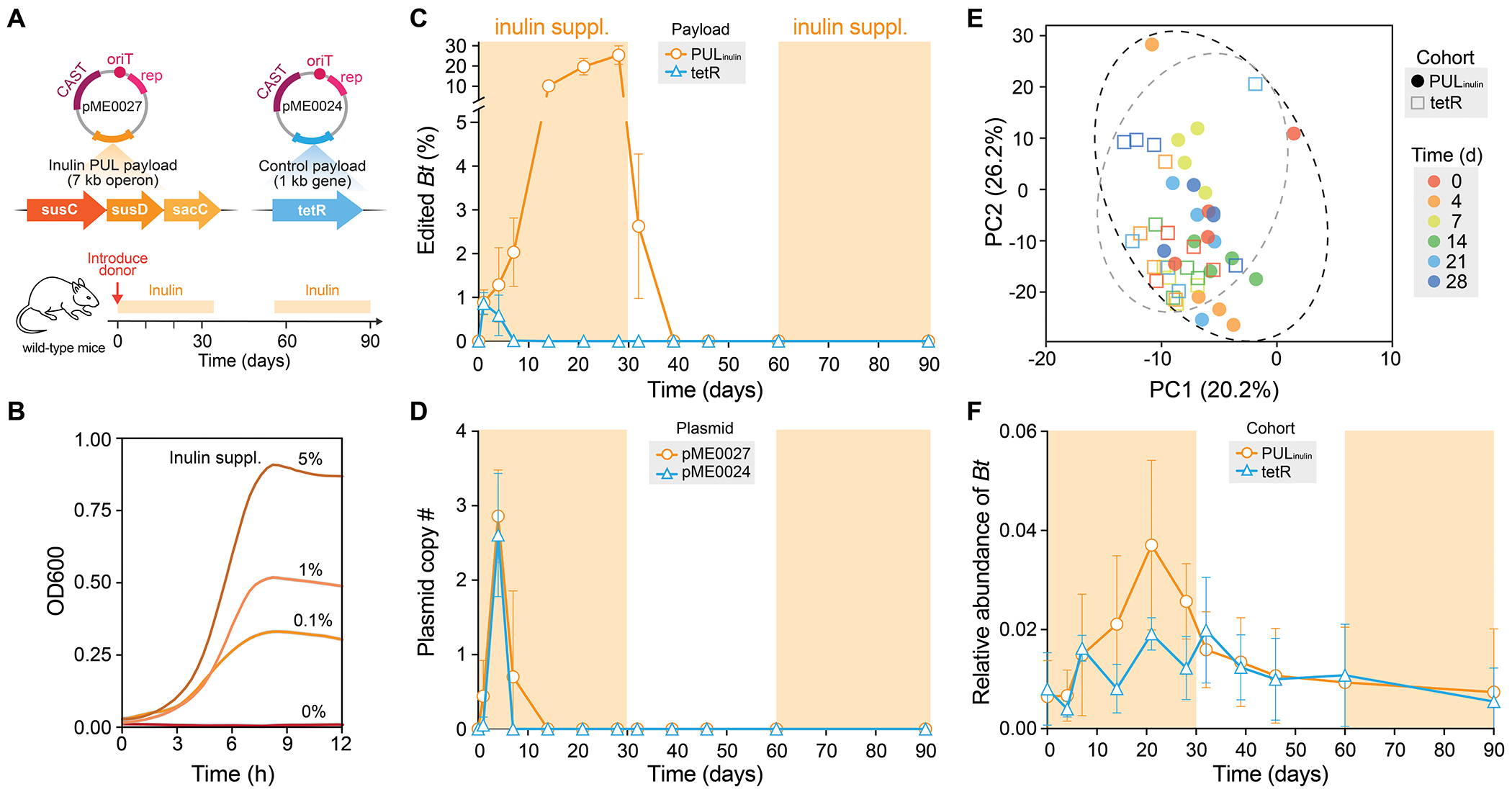
A polysaccharide utilization loci (PUL) payload enables controlled colonization of *Bacteroides* by dietary selections in mice. (**A**) *In vivo* MetaEdit experiment targeting native *Bt* with pME vectors encoding PUL_inulin_ or *tetR* control with dietary perturbations. (**B**) Growth curve of PUL_inulin_-integrated *Bt* in minimal media with increasing inulin concentrations. (**C**) Enrichment (%) of edited *Bt* relative to all native *Bt* with PUL_inulin_ (orange) or *tetR* (blue) payloads over time using intermittent inulin supplementation, quantified by qPCR on fecal matter. (**D**) Copy number of pME0024 (tetR, blue) and pME0027 (PUL_inulin_) in *Bt* over time. (**E**) Principle Component Analysis (PCA) of 16S community composition of pME0027-treated (circles) and pME0024-treated (rectangles) mice during inulin supplementation over time. Fill color indicates different time points and dashed circles indicate grouping of cohorts. (**F**) Relative abundance of all *Bt* between PUL_inulin_ or *tetR* cohorts over time. Shaded orange boxes in (C, D, F) indicate time points with 5% dietary inulin supplement. Growth curves and qPCR data in (B–D) are shown as mean ± s.d. for n=3 independent biological or mouse replicates and 16S sequencing data are shown in replicates (n=4) (E) or as mean ± s.d. for n=4 independent mouse replicates in (F).

We next designed a MetaEdit vector (pME0027) encoding a constitutively expressing 7.5-kb inulin PUL payload (PUL_inulin_: SusC, SusD, SacC) from *B. ovatus* (33) for integration into native murine *Bt* (Fig. 3A). Growth assays confirmed that this *Bt* strain does not normally metabolize inulin (Fig. S12A). Integration of PUL_inulin_ into the *Bt glmS* locus (Fig. S12B) resulted in sufficient gene expression to allow growth on inulin as the sole carbohydrate source and not with levan or xylan (Fig. S12C–D). The edited strain grew proportionally with increasing inulin supplementation up to 5% (Fig. 3B). These results confirm that an integrated PUL_inulin_ enables inulin utilization, which can functionally distinguish an engineered strain from wild-type.

We then tested direct *Bt* engineering *in vivo* with the PUL_inulin_ payload and subsequent selective control of edited cells with dietary inulin. Mice were gavaged with a single dose of 10^10^ *Ec^Bt^* donor cells carrying either PUL_inulin_ or a control *tetR* payload (Fig. 3A). Inulin (5% w/v) was provided in the diet immediately following donor gavage for 30 days. Early *Bt* editing was observed for both payloads, but only *Bt-*PUL_inulin_ showed strong selective enrichment, reaching ~30% of the total *Bt* population after four weeks (Fig. 3C). In contrast, *Bt-tetR* peaked at ~1% editing and was quickly lost within four days. We further observed rapid loss of MetaEdit plasmids in *Bt* after one week (Fig. 3D), indicating that long-term persistence occurred only in cells with the genomically-integrated inulin pathway. When inulin was removed from the diet, *Bt*-PUL_inulin_ were quickly lost, and its absence upon reintroduction of inulin after another 30 days suggested that no edited cells remained (Fig. 3C). 16S analysis showed that inulin supplementation did not significantly alter the native flora (Fig. 3E, Fig. S13). While the relative abundance of *Bt* increased slightly during inulin supplementation (~2%) but did not reach statistical significance, the levels returned to baseline after intervention (Fig. 3F).

We next tested MetaEdit’s effect on augmenting strain engraftment by *in vitro* editing a human derived ATCC-*Bt* strain (VPI 5482) then orally administering it as a single dosage (akin to a probiotic) to mice as compared to *in vivo* editing with PUL_inulin_ and *tetR* payloads, respectively (Fig. S14A). The ATCC-*Bt* strain integrated with PUL_inulin_ *in vitro* (Fig. S14B) resulted in similarly sufficient gene expression to allow growth on inulin as the sole carbohydrate source while the WT could not (Fig. S14C). We then orally gavaged three concentrations (10^10^, 10^7^, 10^5^ cells) of ATCC-*Bt-*PUL_inulin_ to separate mouse cohorts compared to the control ATCC-*Bt-tetR* (10^10^ cells) with dietary inulin (5% w/v) supplementation. The highest concentration (10^10^) of ATCC-*Bt-*PUL_inulin_ resulted in robust engraftment, reaching 5% relative abundance during the first week, while the lowest concentrations were below the detection limit (10^5^) or lost comparably (10^7^) to the ATCC-*Bt-tetR* control within 3-4 days (Fig. S14D). We next monitored the engraftment of the high dosage *in vitro* engineered ATCC-*Bt*-PUL_inulin_ compared to *in vivo* MetaEdit within endogenous murine *Bt* with and without inulin supplementation (Fig. S14E–F). ATCC-*Bt*-PUL_inulin_ persisted comparably well to *in vivo* MetaEdit for up to 3 weeks after which it was lost from the microbiome prior to removing inulin from the diet (Fig. S14E–F). These results demonstrate that an inulin-utilization payload enables diet-responsive modulation of both *in vitro* and *in vivo* engineered *Bt*, highlighting MetaEdit’s capacity to directly control modified cells *in vivo* through an orthogonal metabolite.

### Culture-free editing of Segmented Filamentous Bacteria

A number of highly abundant mammalian-associated intestinal bacteria remain difficult to culture *ex vivo* (34), limiting our ability to study and harvest their biological capabilities. A well-known example is Segmented Filamentous Bacteria (SFB), also designated as *Candidatus savagella* (35), which have been an important model commensal microbiota in mice to understand host-microbe interactions (36–38). SFB exhibits strict host dependence and preferentially colonizes the terminal ileum, where it attaches and directly interacts with the intestinal epithelium (39). SFB possess a number of immunomodulatory functions, including activation of intestinal epithelial cells, induction of IgA, and generation and differentiation of antigen-specific tissue-resident CD4^+^ T helper 17 (Th17) cells (36, 38, 40–42). In addition, SFB or SFB-induced Th17 cells provide protection from mucosal or systemic infection and metabolic disease (43–45). Despite its importance, SFB is difficult to culture and has remained genetically intractable, hindering further mechanistic studies.

**Fig. 4:**
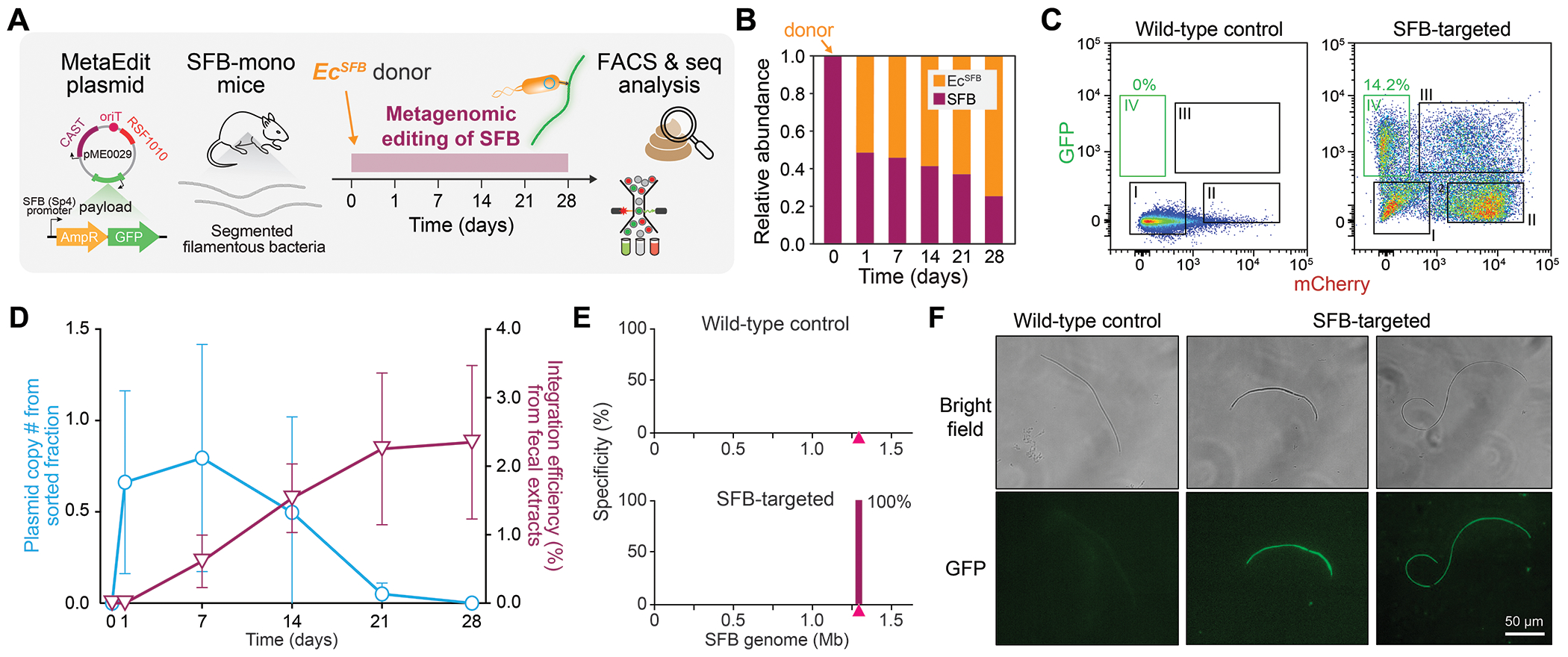
MetaEdit of Gram-positive Segmented Filamentous Bacteria (SFB). (**A**) A schematic of MetaEdit vector and experimental design for SFB engineering. (**B**) Average 16S community composition of SFB-colonized mice treated with *Ec*^*SFB*^ donor over time. (**C**) FACS plots of mCherry and GFP channels from wild-type (no donor) or SFB-targeted mice (*Ec*^*SFB*^ donor). Inner boxes indicate gating for specific cell populations. The four gates represent I) unedited wild-type SFB, II) *Ec*^*SFB*^ donor with lost pME0029 vector, III) *Ec*^*SFB*^ donor with pME0029, and IV) edited SFB expressing GFP. (**D**) Integration efficiency of AmpR-GFP payload into SFB based on fecal analysis (magenta) and copy number of pME0029 vector in sorted SFB cells (blue) over time. (**E**) Genome-wide integration specificity quantified by deep sequencing in (gate I+IV)-sorted SFB cells from (C). (**F**) Bright field and fluorescence images of (gate I+IV)-sorted SFB populations, showing GFP^+^ SFB filaments in edited conditions. 16S and specificity data are shown by a representative replicate in (B, E) and qPCR data in (D) are shown as mean ± s.d. for n=3 independent mouse replicates.

To address this challenge, we developed an SFB-targeting MetaEdit vector (pME0029) that encodes SFB-specific regulatory elements driving CAST machinery and a dual-reporter payload containing ampicillin-resistance (*ampR*) and GFP genes (Fig. 4A). Genomic integration of the GFP payload is achieved with a megRNA guided to an intergenic site downstream of *glmS* in the SFB genome. We optimized MetaEdit by using a mCherry-tagged non-auxotrophic donor *E. coli* (EcGT1) to promote *in vivo* colonization and allow fluorescence-activated cell sorting (FACS) to distinguish donor cells from edited SFB. Donor cells express both mCherry and GFP (mCherry^+^GFP^+^) from the pME0029 vector or only mCherry (mCherry^+^GFP^neg^) once the plasmid is lost/transferred. Since pME0029 is not expected to propagate in SFB without selection, any SFB showing GFP expression (mCherry^neg^GFP^+^) is attributed to MetaEdit. SFB have a long filamentous morphology making them easily distinguishable from *E. coli* donors by flow cytometry based on cell size differences (46).

Germ-free (GF) animals were first mono-colonized with SFB by oral gavage. After 7 days, SFB-mono cohorts we gavaged with either: (A) an *E. coli* donor (*Ec^SFB^*) harboring pME0029 with the GFP payload and SFB-targeting megRNA, (B) a donor (*Ec^ctrl^*) containing a control vector (pME0030) with only CAST machinery but no payload or megRNA, or (C) a vehicle control without any donor cells (Fig. S15A). Fecal sequencing analysis demonstrated robust donor colonization after 28 days (Fig. 4B, Fig. S15B). Next we used a density gradient (46) to isolate long SFB filaments from feces of animals co-colonized with SFB and the donor strain for 28 days and performed FACS-gated on GFP and mCherry channels to discern reporter expression (Fig. S15C–D). Four distinct cell populations could be identified (Fig. 4C), corresponding to wild-type SFB (gate I: mCherry^neg^GFP^neg^), *Ec^SFB^* donor with lost pME vector (gate II: mCherry^+^GFP^neg^), *Ec^SFB^* with the pME vector (gate III: mCherry^+^GFP^+^), and edited SFB (gate IV: mCherry^neg^GFP^+^) (Fig. S15E–H). Strikingly, we detected a high proportion of GFP-expressing SFB filaments (14.2%, mCherry^neg^GFP^+^) (Fig. 4C).

We assessed the overall SFB editing efficiency by PCR on fecal extracts for the integration junction, which showed increasing payload integration over time, reaching 2.3% of all SFB by Day 28 (Fig. 4D, Fig. S16). pME0029 was lost in mCherry^neg^GFP^+^ SFB over time while the integrated GFP payload remained stable (Fig. 4D). Deep sequencing further confirmed 100% on-target integration to the *glmS* site in SFB (Fig. 4E). GFP^+^ SFB filaments were visually identified from sorted populations in *Ec^SFB^* donor treated mice by fluorescence microscopy, but not in donor-negative control mice (Fig. 4F, Fig. S17). We surmise that efficient *in vivo* editing of SFB was enabled by using an engrafting donor strain in germ-free mice, followed by targeted enrichment via Nycodenz density gradient centrifugation (see Methods) and sorting of long, GFP-positive filaments—capturing rare transconjugants distributed along the entire SFB structure. Collectively, these experiments establish the first successful genetic modification of SFB in the murine gut.

## DISCUSSION

The gut microbiome plays fundamental roles in human health and disease, but direct *in vivo* manipulation of bacteria to dissect these roles with the precision of reverse genetics has been challenging. Our results showcase a new metagenomic editing (MetaEdit) strategy that enables *in vivo* microbiome engineering in the gut with high specificity, targeting specific genomes of strains (megabase scale) among thousands of discrete resident species and a collective metagenome size amounting to billions of base pairs. Using human and murine microbiota, we optimized MetaEdit across key gut species, developed methods for sequence-defined strain isolation, and demonstrated that an edited *Bacteroides* strain could be reversibly enriched over a month timescale through dietary inulin supplementation by integrating a 7-kb inulin metabolism operon. A key innovation is culture-free genetic modification of microbes that are difficult to isolate or purify, as highlighted by our ability engineer SFB for the first time — an important immunomodulatory commensal that resides in the small intestine. Collectively, these advances open new avenues for genetic investigations of important processes at the host-microbe interface, such as mucosal immunity or the gut-brain axis (47).

Nevertheless, future efforts will be needed to further expand the reach of MetaEdit to other challenging bacterial communities and environments by overcoming existing limitations relating to vector delivery, DNA integration, and payload persistence. Endogenous barriers against horizontal gene transfer are widespread, including diverse restriction modification (R-M) systems that cleave foreign DNA based on sequence-specific motifs or other defense systems such as CRISPR-Cas (48, 49). Future MetaEdit vectors could be enhanced to eliminate R-M target sequences (50), mimic host methylation patterns (51), or encode defense system inhibitors (52). We observed fitness costs associated with constitutive CAST expression (Fig. S10) and thus envision future versions of MetaEdit exploiting non-replicative vectors (20) for short-term and rapid DNA integration kinetics, alongside improved CAST variants from directed evolution (53) and CAST homolog screening efforts (54, 55). Last but not least, the long-term persistence of integrated payloads will be further facilitated by continued exploration of dietary supplementation (56–58) to enrich and select desired phenotypic traits, while also comprehensively monitoring off-target effects through temporal metagenomics analyses. Importantly, careful attention to biocontainment will be critical to restrict unintended horizontal dissemination of MetaEdit components in future applications, which we propose to achieve using kill switches and/or engineering auxotrophy (59).

MetaEdit constitutes a new approach to embed desired biological functions directly into native microbiota that are already adapted to their environment. This strategy provides an alternative solution to non-native probiotics that are commonly used for microbiome interventions, which suffer from chronic challenges around long-term engraftment (7, 8, 60), and the ability to control payloads with orthogonal dietary supplements enables reversible microbiome engineering (61). Tuning the colonization dynamics of specific natural flora such as *Akkermansia*, *Lactobacillus*, and *Enterococcus* with MetaEdit will further expand editing to different gut compartments, such as the small intestine, and potentially amplify their beneficial effects (62). We envision these strategies leading to more robust and versatile microbiome-based diagnostics and therapeutics (63–67), particularly with the payload flexibility allowing for insertion of individual genes, genetic circuits, or large operonic pathways. Additionally, library-scale gene disruptions (68)) could be achieved by coupling edits to fitness-enhancing payloads within complex microbiotas, allow fundamental mechanistic studies of gene function (20, 69). MetaEdit can be integrated with emerging tools in biosensing (66, 67, 70), expression control (71–73), spatiotemporal profiling (74–76) or antimicrobial development (77). Together, advances in MetaEdit will accelerate how we harness microbiomes for translational medicine and basic discovery.

## MATERIALS AND METHODS

### Plasmid construction

All constructs used in this study with CAST were cloned using all-in-one pSPIN vector orientations described previously (19, 24) and subsequently named Metagenomic Editing plasmids (pME). These constructs were derived from a Type I-F CAST system from *Vibrio cholerae* Tn6677 (VchCAST) (55). Cloning was performed using a combination of Gibson assembly, around-the-horn (inverse) PCR, or restriction digestion and ligation. All DNA fragments used for cloning were PCR amplified using Q5 DNA polymerase (NEB).

To generate a pME vector capable of being maintained in our donor and recipient *Bacteroidaceae* we combined elements from multiple plasmids for seamless replication and selection in both species. Built on a pUC19 backbone, the pME vector carries the pMB1 origin and ampicillin resistance for *E. coli*, while *Bacteroidaceae* compatibility comes from the pTIO-1 origin for replication (78) and erythromycin resistance for selection. A TetX1-based tetracycline resistance gene within the CAST payload enhances selection efficiency. This choice was based on its lower prevalence in native strains compared to *TetQ* and its higher minimum inhibitory concentration (MIC), facilitating more efficient selection in native isolates. CAST, megRNA, and payload expression are tightly regulated by *Bacteroidaceae*-optimized promoters (p^σ70^, p1, p^cfxa^) and fine-tuned RBS of varying strengths to regulate stoichiometry (71, 79, 80). The gRNA was expressed separately downstream of the CAST system under the control of the p1 promoter (derived from 16S rRNA gene in *Bacteroides thetaiotaomicron* VPI 5482) to prevent self-targeting. These promoters were chosen due to their previous characterization for robust expression . The RBS strengths were selected to maintain proper stoichiometry of CAST components: the RBS1 driving *tnsA* was the strongest, followed by RBS2 upstream of *tnsB*, which was slightly weaker. The RBS3 for *tnsC* was weaker than both RBS1 and RBS2. Additionally, *tniQ* was driven by RBS1, *cas8* by RBS2, *cas7* by RBS3, and *cas6* by a strong RBS *pbT1311* (71). *Bacteroidaceae* optimized pME vectors were cloned using synthesized gene fragments and Gibson assembly (TWIST Biosciences and Genscript). Spacers were cloned using oligo duplexes and ligation as previously described (19, 24). A similar strategy was developed for SFB-targeting pME by replacing regulatory elements with SFB promoters and the payload with an ampicillin resistance-GFP dual reporter.

All primers and DNA oligos used in the study were ordered from IDT and provided in Table S1. Cloning was performed using NEB Turbo competent *E. coli* cells, and plasmids were extracted using Qiagen Miniprep columns. The cloned constructs were verified by Sanger sequencing (GENEWIZ) or whole plasmid sequencing by Oxford Nanopore (Plasmidsaurus). For conjugation assays, plasmids were transformed into EcGT1 or EcGT2 *E. coli* donor strains as previously described (21). All *E. coli* strains were grown in LB agar plates or liquid LB media. pME constructs were grown in 100 μg/mL carbenicillin or kanamycin and if in EcGT2, supplemented with 50 μM DAP. All plasmid sequences and their descriptions are available in Table S2.

### Culturing, *in vitro* conjugation, and *in vitro* integration assays

Aerobic transposition assays were performed in *E. coli* BL21(DE3) cells based on a method described previously (18, 19, 24, 81), using plasmid systems composed of pME. Chemically competent cells were transformed with pME, plated, and grown at 37 °C for 16-20 h in the presence of appropriate antibiotics (100 μg/mL carbenicillin). Cells were scrapped and resuspended in 1x PBS. 2 x 109 cells were used for genomic extraction using Wizard Genomic DNA Purification Kit (Promega). All *E. coli* transposition experiments were performed in N = 3 biological replicates.

Anaerobic transposition assays were performed in various human *Bacteroidaceae* previously isolated in a biobank (28) using a conjugative assay previously described with pME plasmids.(21, 24, 82) Briefly, EcGT2 donor competent cells were transformed with pME in the presence of appropriate antibiotics (100 μg/mL carbenicillin) and 50 μM DAP. Unless otherwise noted, bacterial cultures were grown in one-half-diluted Gifu Anaerobic Medium Broth, Modified (mGAM, HyServe 05433) media prepared according to manufacturers’ instructions. Before experimentation, all mGAM media were reduced for 24h under anaerobic conditions (5% H2, 10% CO2, 85% N2) within a Coy Laboratory Products anaerobic chamber. Cells were then scrapped and resuspended in 1x PBS and treated for gDNA extraction as described above. All monoclonal isolates were Sanger sequenced (Azenta Life Sciences) at the 16Sv4 region pre- and post-experimentation to confirm strain identity. All anaerobic transposition experiments were performed in N = 3 biological replicates. Natural isolates used in this study are available from Dr. Harris Wang upon request.

Before conjugation, donor strains harboring conjugative *Bacteroidaceae* expression vectors were grown from a single colony in 5ml of LB-Lennox media (BD) supplemented with 50 μg ml−1 of carbenicillin and 50 μM DAP at 37 °C overnight (~10–16h). We prepared donor and recipient *Bacteroidaceae* cells and carried out conjugations as previously described (24) under anaerobic conditions. Transconjugant colonies were Sanger sequenced at the 16SV4 region to confirm strain identity, and stable plasmid maintenance was confirmed with colony PCR using the primers listed above. Positive *Bacteroidaceae* transconjugants were then picked into 5 ml one-half mGAM supplemented with 20 μg ml−1 erythromycin or tetracycline and grown overnight. These strains were banked in glycerol stocks (25% glycerol final concentration) and stored at −80 °C. Subsequent growth curve experiments were done using overnight cultures grown from these glycerol stocks in one-half mGAM.

### PCR and qPCR analysis of integration

For all *in vitro* transposition experiments (aerobic and anaerobic), all colonies on plates were scraped and resuspended in 1x PBS. To prepare cell lysates, approximately 3.2 × 10^8^ cells (equivalent to 200 μL of culture at OD_600_ = 2.0) were transferred to a 96-well plate. Cells were pelleted by centrifugation at 4,000 *g* for 5 min and resuspended in 80 μL of H_2_O. Next, cells were lysed by incubating at 95 °C for 10 min in a thermal cycler. The cell debris was pelleted by centrifugation at 4,000 *g* for 5 min, and 10 μL of lysate supernatant were removed and serially diluted in H_2_O to generate 10- and 100-fold lysate dilutions for PCR and qPCR analyses, respectively.

PCR reactions were performed using primers annealing to the integration junction within target genomes. Each 25 μL PCR reaction contained 2× Q5 Master Mix (NEB), 0.5 μM of each primer, and 1 μL of 10-fold diluted lysate. Thermal cycling conditions included DNA denaturation (94 °C for 30 s), 30 cycles of amplification (denaturation: 98 °C for 15 s, annealing: 59-68 °C for 15 s, extension: 72 °C for 15 s), followed by a final extension (72 °C for 2 min). Products were resolved by 1-2% agarose gel electrophoresis and visualized by staining with SYBR Safe (Thermo Fisher Scientific). Quantitative PCR (qPCR) was performed in a 10 μL reaction that contained 5 μL SsoAdvanced^™^ Universal SYBR Green Supermix (BioRad), 1 μL H_2_O, 2 μL of primer pair at 2.5 μM concentration, and 2 μL of 100-fold diluted lysate. Two primer pairs were used: (1) T-RL integration products were specifically captured using a forward primer annealing to 5′ upstream to the target site flanking sequence and reverse primer in the CAST payload; (2) a reference gene (*rpoB*) was amplified with primers annealing to genome of the target bacteria. Transposition efficiency (%) was calculated using the formula: transposition efficiency (%) = 2^−ΔCq^*100%, whereΔΔCq = ΔCq(Cq_sample_(T-RL integration)-(reference gene)). Primer sequences are provided in Table S1.

### megRNA design and cloning

megRNAs were designed with 32-nt spacers targeting genomic sites, for isolates or within microbiome targets, with a 5′ CN Protospacer Adjacent Motif (PAM), as previously determined for Vch Type I-F CAST (18, 19). A computational pipeline was developed to assess all possible gRNAs within a given metagenome. In brief, MAG fasta input is parsed into all possible 34 base pair *k-mers* and these are then filtered for *k-mers* containing a 5’-CN PAM. *k-mers* are then mapped back to MAGs for counts with BLASTn (83) at 100% sequence identity and split into two lists containing either multi-mapping spacer candidates or single-mapping spacer candidates. Minimal off-targets were then assessed by mapping back the spacer sequence to the available (meta)genomes in a secondary module. Specifically, the Bowtie2 alignment tool (84) was used to evaluate each spacer candidate for potential genome-wide off-targets. Spacers are considered to have potential off-targets when Bowtie2 detects alignments exhibiting lower than a user-specified maximum mismatch limit. For bacterial genomes, we find that this process usually results in a sufficient number of spacers within each window, without the need for scoring each spacer candidate. For Type I-F Cascade spacers (such as VchCAST), the program converts flexible bases—those bases occurring every 6th position, which do not contribute to spacer-protospacer complementarity within the R-loop to ‘N’ to exclude these bases from contributing to the mismatch count for the genome-wide off-target search. Spacers with significant potential off-target matches are removed resulting in a final list of megRNA candidates.

Each construct containing a spacer was first generated with a filler sequence containing tandem BbsI or BsaI recognition sites in place of the spacer. New spacers were then cloned into the array by phosphorylating complementary oligonucleotides with T4 PNK (NEB), hybridizing the oligonucleotides and ligating them into the BsaI- or BbsI-digested plasmid. All spacer sequences used for this study are available in Table S3.

### Animal ethics statement

All animal experiments were performed in compliance with Columbia University Medical Center IACUC protocols AC- AABK9603.

### *In vivo* conjugation assays in mice

Conventionally raised gnotobiotic mice or C57BL/6 female mice (Jackson Laboratories) were used throughout the study. Gnotobiotic experiments were conducted at the Microbiome Translation Center at Mount Sinai with Dr. Jeremiah Faith following their well-established core facility procedures. All native microbiome experiments in Jackson mice were conducted at Columbia University following IACUC guidelines. All studies contained at least three to five mice per cohort. To equilibrate the mouse gut microbiome ahead of time, we mixed mice from multiple litters, cohoused them for at least 1 week before all experiments, and randomly allocated them into groups. To standardize the microbiome in early MetaEdit experiments (Fig. 2), we developed a native mouse model using FMT from Jackson mice with high *Bacteroidaceae* levels, creating one-shot glycerol stocks from fecal samples for gavage, which stabilized the microbiome within two days and peaked in *Bt* abundance by days five to six. This method was not used for subsequent experiments with inulin or SFB.

For *in vivo* conjugation, donor strains of EcGT2 harboring the appropriate pME vector were grown overnight and washed the following day with 1x PBS. Mice were then gavaged at least once with 10^9^ donor cells in 200 μl of PBS at 8–12 weeks old. Control mice included either a NT pME donor or PBS gavage. Fecal matter was collected immediately before gavage and periodically after gavage for analysis of the resulting microbiome populations by PCR/qPCR, Tn-seq, 16S/metagenomic sequencing, plating, FACS, and microscopy. Upon completion of the study, mice were euthanized.

Conjugation efficiency was determined by adapting an assay previously described (21, 24, 28). Briefly, fecal matter was homogenized (see Methods: Isolation of targeted gut bacteria from mice) in an anaerobic chamber (Coy labs) and serial dilutions ranging from 10^0^ – 10^−8^ were plated on one-half-mGAM (Hyb) supplemented with the appropriate antibiotic to select for the pME backbone marker (i.e., erythromycin). For normalization, the sample fecal dilutions were plated on non-selective mGAM and the resulting calculation for conjugation efficiency = (# colonies on selective media)/(# colonies on non-selective media). Donor relative abundance was determined by metagenomics. Our specificity for detecting the donor *E. coli* with metagenomics is 0.001 relative abundance (0.1%) at our average library coverage (5 million reads per library).

### DNA extractions from isolates and microbiome

gDNA of isolates were extracted in 96-well format using a silica bead beating-based protocol adapted from a prior study.(28) To begin, 200 μl 0.1 mm Zirconia Silica beads (Biospec, 11079101Z) and 300 μl lysis solution (50 mM Tris–HCl, pH 7.5 and 0.2 mM EDTA) were added to each well of 96-well deep-well plates (Thermo Fisher Scientific, 07-202-505). Next, 200 μl culture solutions (OD = ~1.0) of isolates were added to each well and the plates were centrifuged for 5 min at 4,500g and affixed with a sealing mat (Axygen P-DW-20-C). To avoid overheating during bead beating, the plates were vortexed for 5 sec and incubated at −20 °C for 10 min before beating. Then, plates were fixed on a bead beater (Biospec, 1001) and subjected to bead beating for 2.5 min, followed by a 5-min cooling period. The bead beating cycle was repeated for a total of three times with a final cooling of 30 minutes and plates were centrifuged at 4,500g for 10 min to spin down cell debris. Next, 60 μl cell lysate was transferred to a 96-well PCR plate (Bio-Rad, HSP3801) and 15 μl proteinase K solution (50 mM Tris–HCl, pH 7.5 and 1 μg/μl proteinase K (Lucigen, MPRK092)) was added. Finally, the cell lysate was subjected to proteinase K digestion on a thermal cycler (65 °C 30 min, 95 °C 30 min, 4 °C infinite) and bead-cleaned to purify the DNA using Ampure Beads (Agilent) at a 1x ratio. DNA was stored at −20 °C or used directly for various assays. gDNA extraction from bulk fecal samples (10-20 mg) were performed using the same protocol in 96-well format.

### Quantification of site-specific integration efficiency *in vivo*

PCR and qPCR-based methods described above for transposition efficiency in isolates were applied to native targets within the gut microbiome following the same protocol but utilizing gDNA extracted from fecal matter as input (2 u\μL ranging from 1-10 ng). Primer pairs for T-RL were used with a reference gene as determined from previously assembled metagenomic assemblies from Jackson mice microbiomes.

### Quantification of plasmid copy number

Plasmid copy number in isolates and metagenomes was determined using qPCR-based methods described above for transposition efficiency, but changing the integration junction primer set for a pME backbone primer set (Table S1). Copy number normalization was conducted in a similar fashion as transposition efficiency via a house keeping gene without the multiplication by 100% (i.e., plasmid copy (#) = 2^−ΔCq^).

### Deep sequencing library preparation and sequencing for integration

We adapted a tagmentation-based Tn-seq protocol previously described to sequence all insertion events within target isolate genomes or metagenomes (85). 1-10 ng of extracted DNA from either isolate or microbiome transposition assays were mixed with 1.25 μL of tagmentation DNA buffer (Illumina) and 0.25 μL of tagmentation enzyme mix (Illumina) in a final volume of 2.5 μL and incubated at 55°C for 10 minutes. The tagmentation reaction was quenched, and DNA was amplified by a 20-cycle PCR-1 step using a CAST payload-specific primer along with a tagmentation-specific primer at an annealing temperature of 59°C. After amplification, PCR-1 was cleaned up from leftover primers with Ampure beads at a 1.5x ratio, eluted in 12 μL, and 2 μL were used as a template for a nested PCR-2. PCR-2 used a primer specific to the right transposon end, having a universal Nextera adaptor (i5) overhang, and a reverse primer using a Nextera adaptor (i7) that was specific to tagmentation (0.42 μM) (Table S1). Y3 mix is a combination of three primers with variable length between the CAST transposon-end specific region and the universal Nextera adaptor in order to introduce diversity during Illumina flow cell clustering (Table S1). After 15 cycles of amplification at an annealing temperature of 54°C, the amplified DNA was purified using 1.5x magnetic beads and eluted in a final 20 μL volume. Both PCR-1 and PCR-2 conditions utilized 30 μL of KAPA Hifi master mix (Roche) to a final reaction volume of 60 μL. The final barcoded DNA library was resolved by 2% agarose followed by excision and isolation within the size range of 300-600-bp using the Monarch Gel Extraction Kit (NEB). Deep sequencing libraries were pooled and quantified by the HS dsDNA Qubit kit (Thermo Fisher). Sequencing was performed on Illumina MiSeq or NextSeq platforms with the NextSeq high-output kit. 75/150-cycle single-end reads were obtained, and automated adaptor trimming and demultiplexing (Illumina) were performed.

### Analysis of deep sequencing data for integration

Analysis of deep sequencing Tn-seq data was performed using a custom Python pipeline as described (19). Demultiplexed raw reads where half of the bases had a Phred quality score of less than 20, which corresponds to greater than 1% base miscalling were removed from the analysis. Reads containing the last 20 bases of the right transposon-end sequence (5′ TGTTGATACAACCATAAAAT 3′) and a 17-bp adjacent flanking genomic sequences were extracted and noted as the total-transposon-end containing reads. This 17-bp adjacent region represents the fingerprint region used to identify transposon insertions and was used to align to the reference genome or plasmid using Bowtie2 (84). The reference isolates genomes were generated from a biobank from a recent publication(28) and metagenomes for Jackson mice were sequenced and assembled in-house. Only reads which mapped perfectly and only once to the genome were chosen. Reads which did not get mapped to the genome were checked for sequences corresponding to the pME vector and noted as Donor contamination. Alignments from Bowtie2 were used for generating genome/plasmid-wide coordinates for integration. If the read was mapped to the same strand as the input fasta file, then it was noted as on ‘fwd’ strand, and if mapped to the complementary strand as the input fasta file, then it is noted as on ‘rev’ strand. The read ‘position’ was indexed at the fifth position of target site duplication (TSD) for each event, with respect to the ‘fwd strand’. The orientation of the integration relative to the fasta file was concluded based on whether the library was sequenced from the right end or the left end of the transposon for TagTn-seq. The orientation of the transposon insertion with respect to the protospacer at the on-target window (100-bp from the end of protospacer), was noted as target-left-right (T-LR) or target-right-left (T-RL). Untargeted reads and on-target reads were assigned using a custom Python script. For genomic/metagenomic integration, reads that mapped within a hundred base window after the end of the protospacer were noted as on-targets, and reads elsewhere in the genome as untargeted. Raw reads for integration at on-target and untargeted sites for each sample were normalized with the total transposon-end containing reads detected for that sample and further scaled with respect to the sample having the highest transposon-end containing reads. In order to minimize amplification biases during deep sequencing only unique insertion sites were used for reporting sequence preferences in transposition. For visualizing normalized plots comparing integration across the genome, raw reads for each coordinate for a sample were normalized and scaled in the same way as above and plotted with Seaborn.

### 16S sequencing and analysis

gDNA extracted above was used as template for PCR amplification of the 16S rRNA V4 region and multiplexed barcoding of samples were done in accordance with previous protocols.(28) Briefly, the V4 region of the 16S rRNA gene was amplified with customized primers that contain: (i) matches to updated EMP 505f and 806rB primers (86) and (ii) use of NexteraXT indices such that each index pair was separated by a Hamming distance of >2 and Illumina low-plex pooling guidelines could be used. The samples were subjected to 16S V4 amplification on a thermal cycler (98 °C 30 s, 40 cycles: 98 °C 10 s, 55 °C 20 s, 65 °C 60 s; 65 °C 5 min; 4 °C infinite). The resulting amplicon libraries were manually pooled and subjected to gel electrophoresis on E-Gel EX Agarose Gels, 2% (Thermo Fisher Scientific, G402002). Expected DNA bands (~390-bp) were excised from gel and extracted by the Monarch Gel Extraction Kit (NEB). Gel-purified libraries were quantified by Qubit dsDNA HS assay (Thermo Fisher Scientific, Q32851) and sequencing was done with the Illumina MiSeq system (300V2 kit).

Raw sequencing reads of 16S V4 amplicon were analyzed by USEARCH v11.0.667 (87). Specifically, paired-end reads were merged using ‘-fastq_mergepairs’ mode with the default setting. Merged reads were then subjected to quality filtering using ‘-fastq_filter’ mode with the option ‘-fastq_maxee 1.0 -fastq_minlen 240’ to only keep merged reads with less than one expected error base and greater than 240-bp. Remaining reads were deduplicated (-fastx_uniques) and clustered into ASVs (-unoise3) at 100% identity, and merged reads were then searched against ASV sequences (-otutab) to generate ASV count table. Taxonomy of ASVs was assigned using Ribosomal Database Project classifier v2.13 trained with 16S rRNA training set 18 (88). Relative abundance of ASVs in bulk samples is defined as reads count of ASVs normalized by total number of mapped reads and plotted with ggplot2 using custom R scripts.

### Isolation of targeted gut bacteria from mice

Fresh fecal pellets were collected from mice, and live gut bacteria were isolated by mechanical homogenization within Coy Laboratory Products anaerobic chamber. All reagents described were degassed in the anaerobic chamber under gas conditions described above. Briefly, 250 μl of PBS was added to previously weighed pellets in a microcentrifuge tube. Pellets were thoroughly mechanically disrupted with a motorized pellet pestle, and then 750 μl of PBS was added. The disrupted pellets in PBS were then subjected to four iterations of vortex mixing for 15 s at medium speed, centrifugation at 1,000 r.p.m. for 30 s at room temperature, recovery of 500-750 μl of supernatant in a new tube, and adjustment of that volume of PBS. The resulting fecal extract of isolated cells were pelleted by centrifugation at 4,000 x g for 5 min at room temperature, the supernatant was discarded, and cells were resuspended in 0.5–1.0 ml of PBS. All gut bacteria isolations were performed by plating a previously determine dilution (via conjugation assay results) on one-half-mGAM media supplemented with the appropriate antibiotic respective to the payload and pME backbone marker (e.g., tetracycline) and grown 24-48 hours. Colonies were screened via PCR directly with integration junction and vector backbone primer sets, and positive hits were grown in 5 mL of one-half-mGAM liquid media supplemented with the appropriate antibiotics for further work. The rest of the colonies on the plate were processed for DNA extract as described above for 16S sequencing.

### Growth curves of bacteria

For all growth experiments with isolates, overnight bacterial cultures were grown in an anaerobic chamber (Coy labs) and were back diluted 1:100 before addition to 96-well plates containing one-half mGAM liquid or *Bacteroides* minimal media (29) totaling to a final volume of 200 μl when combined. Media was supplemented with appropriate antibiotics and carbon source (i.e., inulin) when needed. Cultures were then incubated at 37 °C in shaken media for 24-48 h under anaerobic conditions and OD600 measurements were taken at 30-60 min intervals using an Epoch2 Microplate Spectrophotometer (Agilent Technologies). To determine statistical differences in relative growth between conditions, two-sided independent t-tests with Benjamini–Hochberg correction were performed in R to determine FDR-adjusted P values unless otherwise stated. For detection of microbiome growth capacity in polysaccharides, fecal samples from various mouse vendors (Jackson, Taconic, Charles River) were homogenized as described above and 1/100 dilutions of the fecal extracts were used to seed mGAM and minimal media conditions in 96-well plates. Growth measurements were then conducted the same as described for isolates. Primary data for growth curves are provided in Supplemental Figure S18 (Fig. S18).

### *In vivo* selections for integrated payloads in mice

For studies to select for integrated payloads in target bacteria within the gut microbiome of mice, 5% inulin (PUL payload in *Bacteroides*) dissolved in 400 mL of diH_2_O and filter sterilized through a 0.22 μM filter prior to administration. New supplemented water was changed every 5-7 days. Mice were handled with standard care unless otherwise noted.

### SFB mouse model and conjugation assay

Unless otherwise noted, SFB colonization was performed 2 weeks prior to conjugation by single oral gavage of fecal suspension from SFB-enriched mice as previously described (46) under gnotobiotic conditions to generate mono-colonized mice. To control for variability in SFB levels in feces used for gavage, all gavages were performed with frozen stocks from a single batch of SFB-enriched feces. To generate SFB-enriched feces a single cohort of 10 adult SFB-negative maximum barrier NSG mice from The Jackson Laboratory were colonized with feces from SFB mono-colonized mice. Fecal samples were obtained in the period of 8 weeks, tested for SFB by qPCR and frozen as batch aliquots at −80C. Control SFB-negative feces were collected from a separate cohort of littermate NSG mice housed in a similar manner. SFB colonization levels were confirmed by qPCR and normalized to levels of total bacteria (UNI) as previously described (46).

After establishment of SFB mono-colonized mice, a cohort was gavaged with 10^10^ EcGT1 donor, which is a non-auxotrophic strain to allow for colonization. This donor harbors a pME with SFB promoters driving CAST machinery and a dual reporter payload consisting of an ampicillin resistance (AmpR) gene and sfGFP. As controls, one cohort was gavaged with just the donor (no pME) and a second cohort was gavaged with 1x PBS. All gavage and fecal sampling conditions were conducted as described above.

### FACS measurements for SFB transconjugants

SFB from fresh fecal pellets were analyzed for evidence of successful conjugation using FACS. Fecal matter was collected and homogenized as described above and subjected to a Nycoprep gradient treatment to enrich for SFB, as previously described (46). The enriched SFB solution was processed with a BD FACSAria II cell sorter operated with BD FACSDiva software in order to gate for sfGFP (FITC filter 515/10 nm) and mCherry (mCherry filter 616/26 nm). Initially, a PBS gavaged sample was incubated with Syto9 (1/1000 dilution) for 15 minutes in order to stain all bacterial DNA. This sample was then processed by FACS in order to gate all bacteria separately from the background fecal samples. Next, double-gating on GFP and mCherry channels was used to select for cells with GFP^+^::mCherry^−^ fluorescence. We then gated for longer bacteria (SSC-W) due to the long filamentous morphology previously described for SFB (46). In addition, we took background events into account by using the GFP^+^::mCherry^−^ fluorescence detected in the fecal sample before gavage as the baseline signal. An increase over the baseline signified an enrichment of transconjugants. Population density (cells per gram of fecal matter) was calculated as the number of cells sorted over the mass of the sorted fecal sample. The following gates were determined: 1) donor strain harboring the pME vector (GFP^+^::mCherry^+^), 2) donor strain only (GFP^−^::mCherry^+^), SFB without a transferred vector (GFP^−^::mCherry^−^), and SFB transconjugants (GFP^+^::mCherry^−^). Gates were sorted into separate microcentrifuge tubes with 200-500 μL of PBS. FACS plots were formatted with FCS Express 6.

### Microscopy of GFP^+^ SFB

Sorted bacteria from the FACS measurements described above were directly used for GFP detection in SFB and performed under a microscope (Nikon Eclipse Ti2). All microscopy was done on 10 μL of sample dropped onto a Superfrost Plus microscope slide (Thermo Shandon) and covered with a glass coverslip. Slides were air-dried until the PBS receded from the edges of the coverslip and then were sealed with clear nail polish. Bacteria were imaged at 40x magnification. Observations were done on bright-field to detect bacteria and GFP and RFP channels were then utilized to detect GFP and mCherry expression. Images were captured and processed by Nikon Elements AR software.

### Statistics and reproducibility

Integration/conjugation assays were performed with three independent biological replicates. qPCR and analytical PCRs resolved by agarose gel electrophoresis gave similar results in three independent biological replicates. Sanger sequencing of PCR amplicons was performed once for each isolate. Next-generation sequencing of libraries was performed once. Mice experiments contained multiple cohorts per condition with a minimum of 3-5 independent biological replicate per cohort.

## Supplementary Material

Suppl Material

Suppl Table

## Data Availability

Next-generation sequencing data are made available in the National Center for Biotechnology Information (NCBI) Sequence Read Archive SUB15009166. All custom bioinformatic scripts used for data analysis are available at https://github.com/sternberglab/Gelsinger_et_al_2025. Datasets generated and analyzed in the current study are available from the corresponding authors upon reasonable request.
