# Supplementary material for: Metagenomic editing of commensal bacteria in vivo using CRISPR-associated transposases
Source: Science. Author manuscript; Available in PMC 2026 Mar 10. (PMC12969935; doi:10.1126/science.adx7604)
Supplement: Suppl Material [file NIHMS2146174-supplement-Suppl_Material.docx]

Supplementary Materials for

**Metagenomic editing of commensal bacteria *in vivo* using CRISPR-associated transposases**

**Authors:** Diego Rivera Gelsinger^1,2^, Carlotta Ronda^2,8^, Junjie Ma^3^, Om B. Kar^1^, Madeline Edwards^3^, Yiming Huang^1,9^, Chrystal Mavros^1^, Yiwei Sun^1^, Tyler Perdue^1,4^, Phuc Leo Vo^5,10^, Ivaylo I. Ivanov^3^#, Samuel H. Sternberg^2,6^#, Harris H. Wang^1,7^#

Corresponding authors: [harris.wang@columbia.edu](mailto:harris.wang@columbia.edu), [shsternberg@gmail.com](mailto:shsternberg@gmail.com), [ii2137@cumc.columbia.edu](mailto:ii2137@cumc.columbia.edu).

**The PDF file includes:**

Figs. S1 to S18

**Other Supplementary Materials for this manuscript include the following:**

Tables S1 to S4 (provided as a separate file)

Table S1: Full list of primers and oligos used in the study.

Table S2: Full list of plasmids used in the study.

Table S3: Full list of spacers used in the study.

Table S4: Full list of genetic parts used in the study.

**
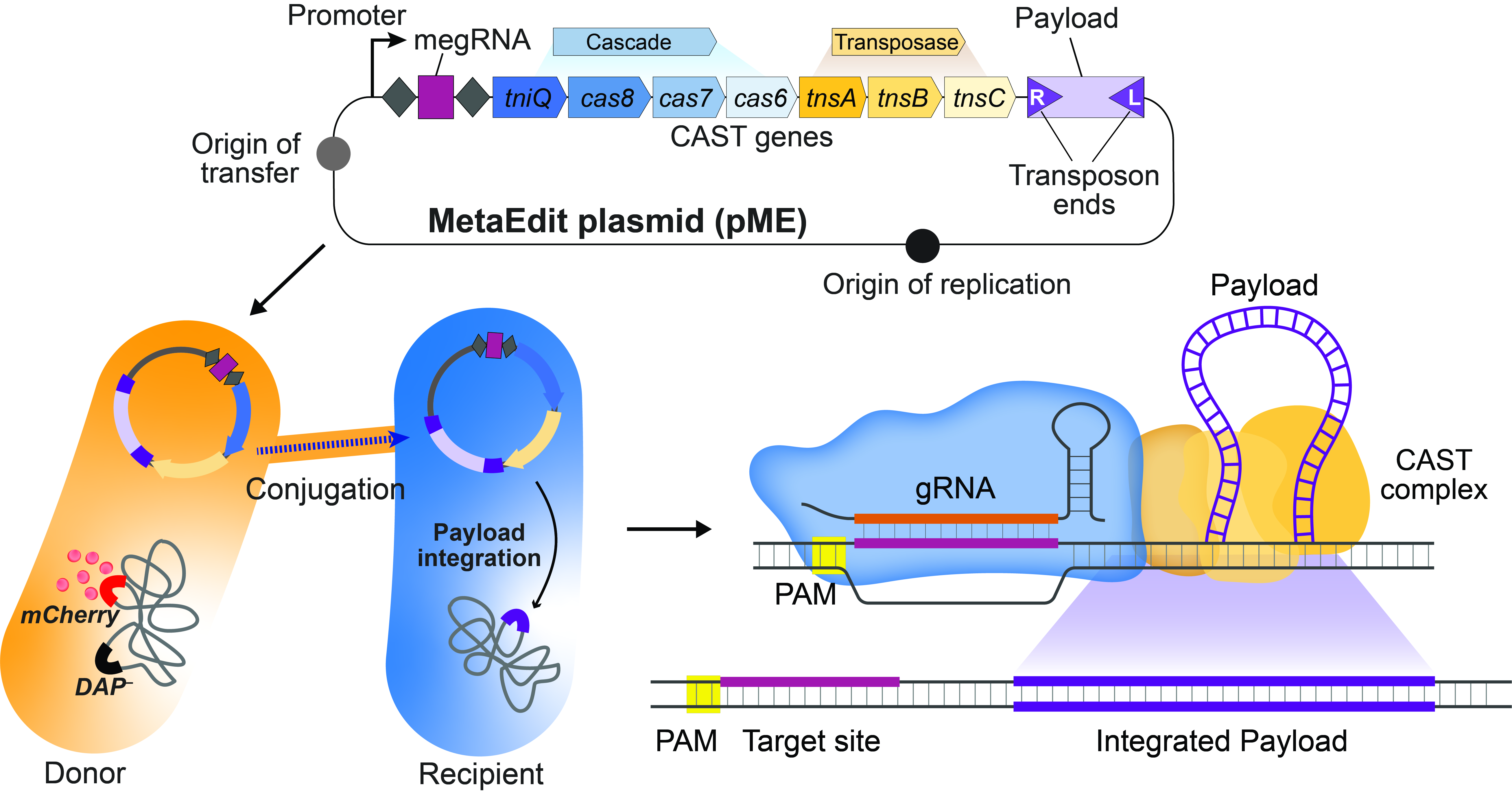
**

**Fig. S1. Schematic of Metagenomic Editing (MetaEdit) technology.** MetaEdit drives precise genetic payload integration in microbiomes via conjugative vectors encoding CRISPR-associated transposases (CAST). The operonic inset (top) shows CAST genes comprised of the Cascade (*tniQ*, *cas8*, *cas7*, *cas6*) and Transposase (*tnsA*, *tnsB*, *tnsC*) modules. Vectors (pME) are highly customizable through traditional cloning to replace regulatory elements, megRNA sequences, payloads, and replication origins. Vectors are transformed into a mCherry-tagged *E. coli* donor that is a DAP auxotroph. Upon donor-target contact, RP4-mediated conjugation transfers the MetaEdit vector (bottom left), and the RNA-guided CAST complex integrates the payload ~49-bp downstream of the target site in the recipient genome (bottom right).


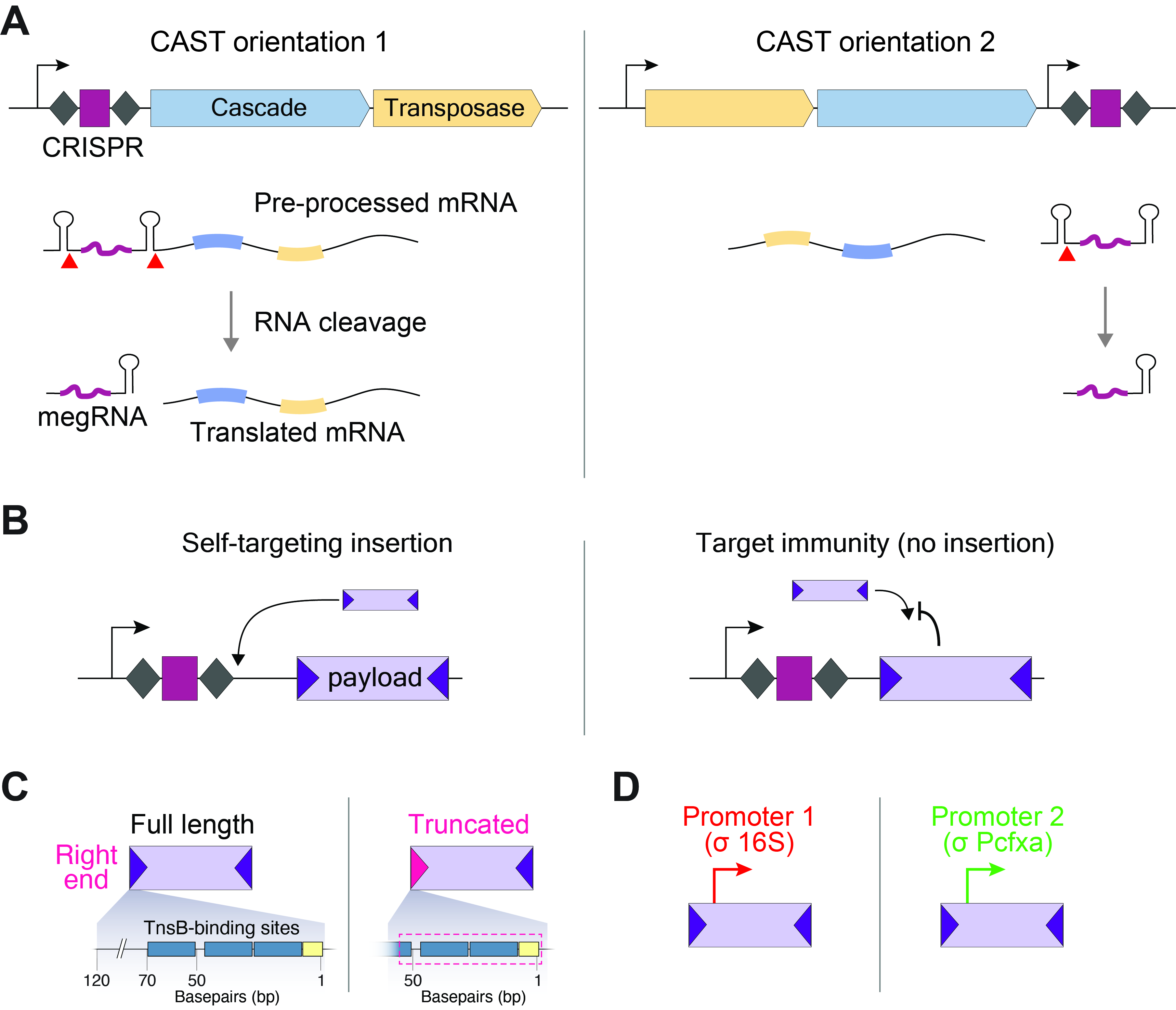


**Fig. S2. Testing optimal arrangements of CAST machinery in *Bacteroidaceae*.** Schematic illustrating the parameters tested for optimal MetaEdit activity in Fig. 1B. (**A**) Testing orientation of CAST machinery arrangements of the Cascade and Transposase gene modules for megRNA generation from the preprocessed mRNA. (**B**) Testing positioning of the CRISPR array relative to the payload to prevent self-targeting insertions. In the case of self-targeting, Cascade targets the spacer sequence within the CRISPR array itself during RNA-guided transposition, resulting in insertion events within the expression cassette that can inactivate the CAST system. To circumvent this undesired self-targeting product, positioning the CRISPR array in close proximity to the payload itself enables protection from self-targeting due to the mechanism of transposon target immunity (*19*). (**C**) Altering the length of payload right transposon end (RE). The full length RE of VchCAST feature repetitive binding sites for the *tnsB* protein in the Transposase module which play a role in integration efficiency. Previous reports show that use of intransposon variants containing a 57-bp truncated RE improves product purity and efficiency (*18*, *19*). (**D**) Testing two previously published *Bacteroidaceae* promoters for optimal payload expression after integration (*71*).

**
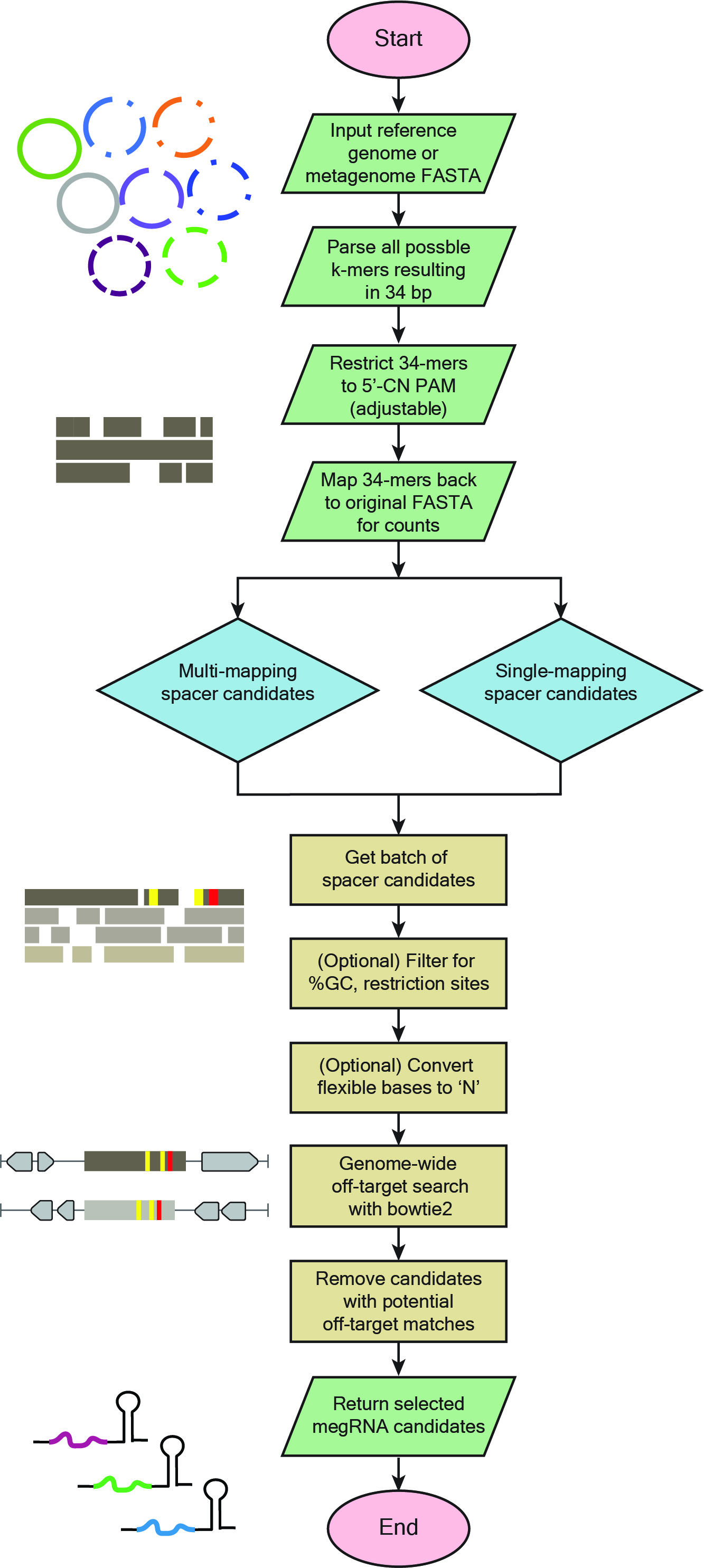
**

**Fig. S3. Computational pipeline for megRNA design.** Logic workflow for computationally designing metagenomic editing gRNAs (megRNAs) from metagenome data as input was adapted from (*19*). Specifically, megRNAs with 32-nt spacers targeting metagenomes were selected based on a 5′-CN PAM, optimized for Vch Type I-F CAST (VchCAST). A metagenome-wide search parsed metagenome-assembled genome (MAG) FASTAs into 34-bp *k-mers*, filtered for PAMs, and mapped these *k-mers* back to MAGs using BLASTn to identify unique or multi-mapping candidates. Off-target potential was evaluated with Bowtie2, excluding hits above a mismatch threshold (>2). Flexible bases in Type I-F spacers were masked to improve specificity. Final megRNA lists should contain only high-specificity, single-mapping spacers.

**
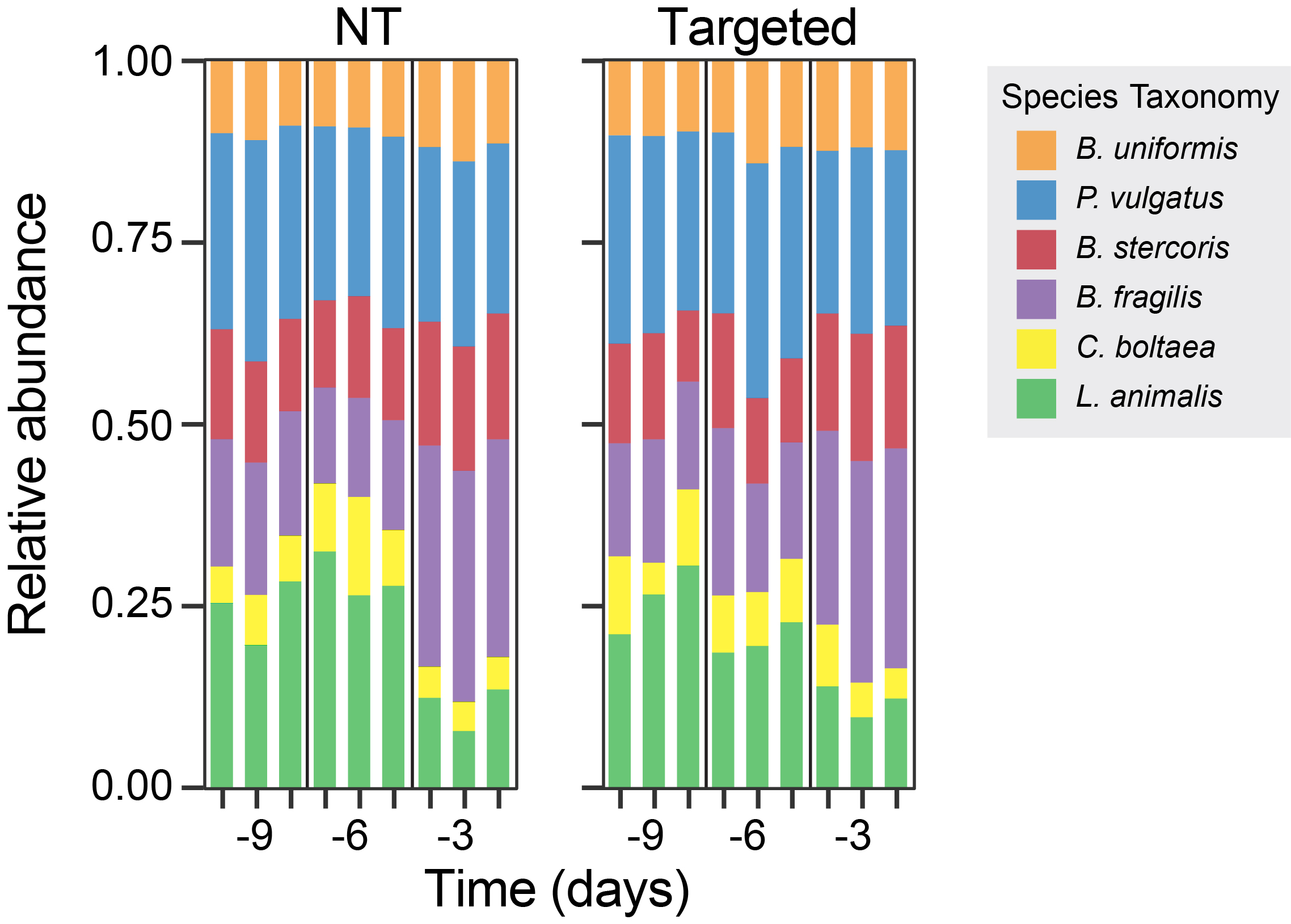
**

**Fig. S4. Colonization of gnotobiotic mice with human gut isolates.** Graph of relative bacterial abundance from 16S rRNA sequencing. Visualized are species-level colonization profiles in gnotobiotic mice gavaged with defined human gut isolates at three time points (9 days, 6 days, and 3 days) before donor gavage (day 0). Deep sequencing 16S data are shown as mice replicates (n=3).

**
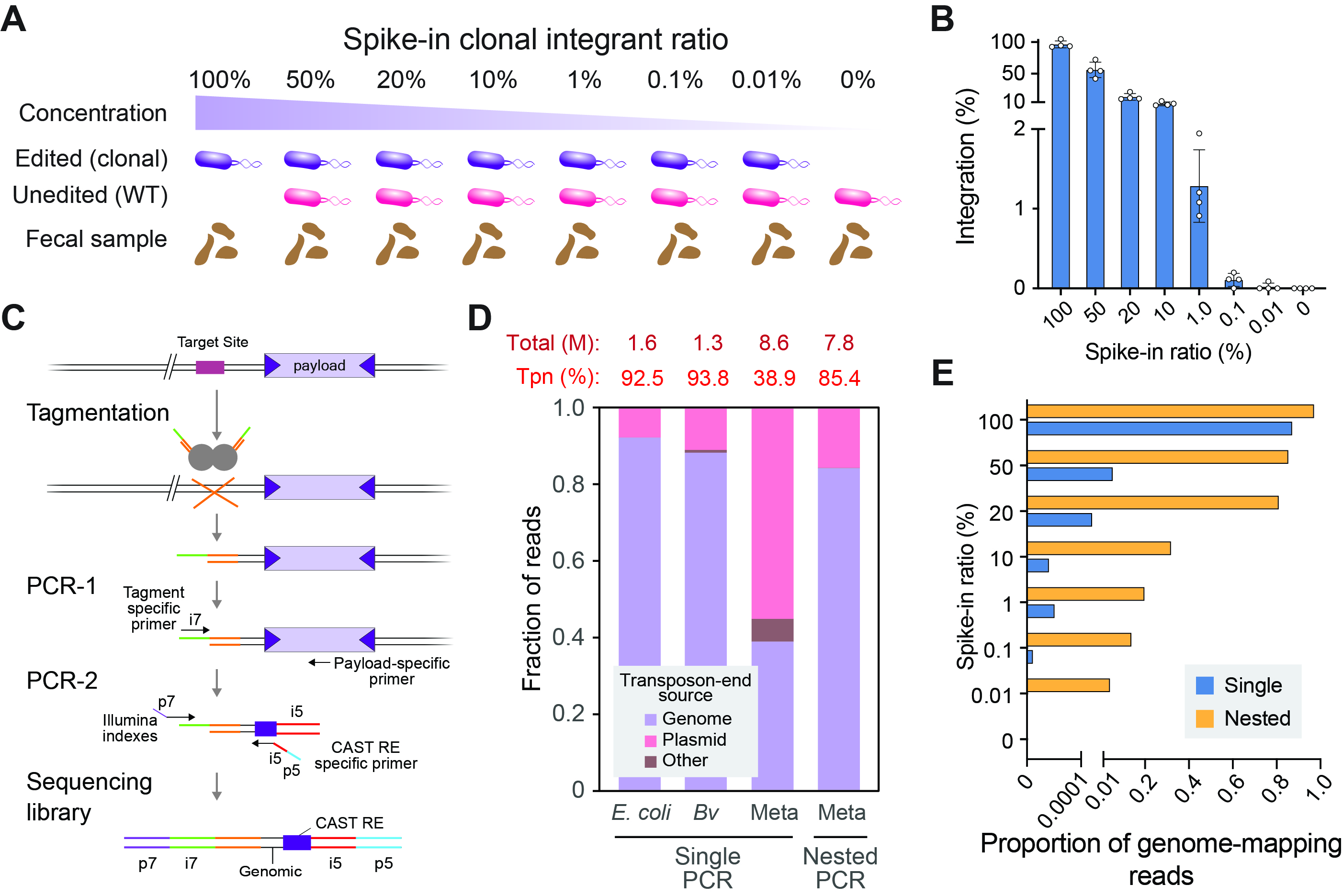
**

**Fig. S5: Parameterizing the detection limit of DNA integration within a complex microbiome.** (**A**) Diagram of the pipeline for determining the detection limit of integration via qPCR and transposon sequencing (Tn-seq). Integration and specificity efficiencies were experimentally simulated within a microbiome by mixing MetaEdit clonally integrated (Edited) and unintegrated (Unedited) *E. coli* isolates in known ratios within a mouse fecal sample harboring a complex microbiome. (**B**) Simulated integration efficiency (%) of the clonal integrant across a titration from conditions in (**A**) as measured by qPCR. (**C**) Diagram of the Tn-seq integration specificity pipeline using tagmentation and nested PCR. Metagenomic DNA is randomly fragmented and tagged with adapter sequences via tagmentation. PCR-1 uses a primer specific to the tagment adapter and a primer specific to the MetaEdit payload. PCR-2 then uses PCR-1 product as template with a primer adding Illumina indexes and a primer specific to the right transposon end (RE) of the CAST integration yielding a final sequencing library. (**D**) Distribution of Tn-seq reads with total number of million (M) reads (Total) and percentage (%) of transposon-end containing (Tpn) reads for each library condition. Based on Tpn reads, in purple are genome-mapping reads that map correctly as an on-target integration event. In pink are contaminating reads from the MetaEdit plasmid which contain an intact RE which allows for PCR events. In brown are other reads that do not map to genome or plasmid. While a single PCR (PCR-1) is sufficient to predominantly capture expected genomic integration events in isolates it is insufficient within metagenomic fecal samples (Meta). Adding a nested PCR (PCR-2) step drastically increases genome-mapping reads in metagenomic samples. (**E**) Detection of genome mapping reads across a titration from conditions in (**A**) using single- or nested-PCR steps as quantified by Tn-seq. Integration efficiency data in (B) are shown as mean ± s.d. for n=3 independent biological replicates and Tn-seq data are shown by a representative replicate in (D-E).

**
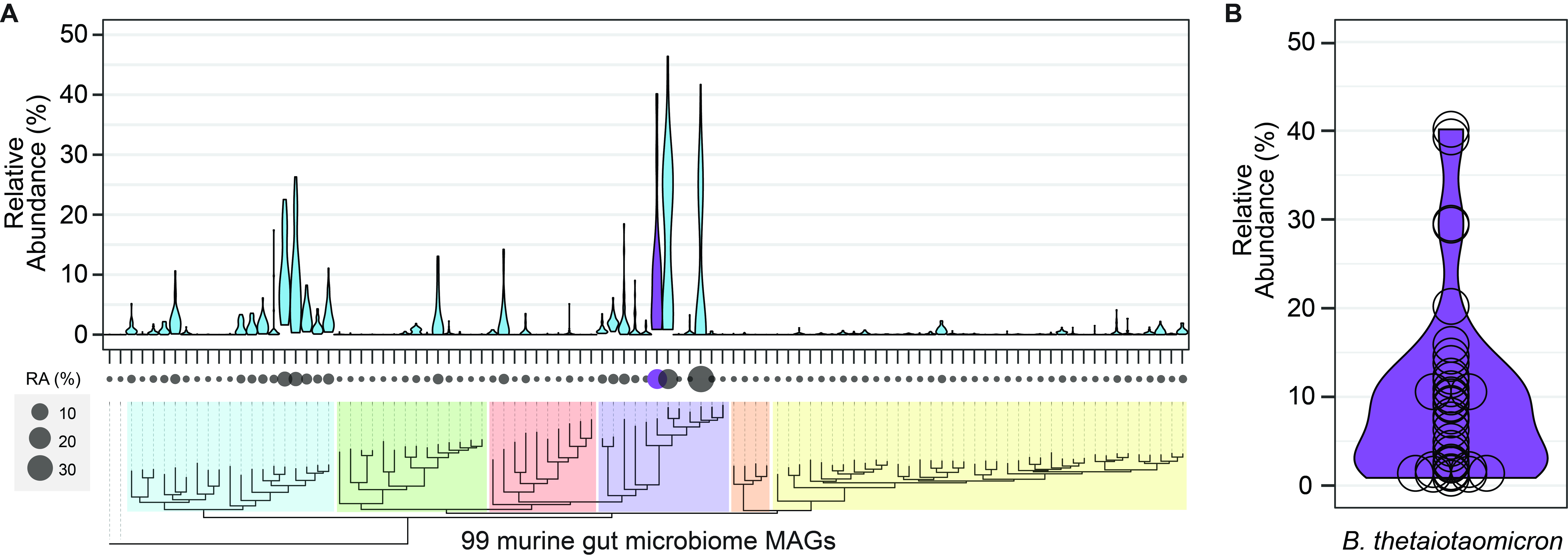
**

**Fig. S6: Distribution of metagenomic relative abundance for each MAG in SPF mice.** (**A**) Visualized are violin plots of the relative abundance for all MAGs ordered phylogenetically as in Fig. 2A. (**B**) Zoomed in view of the violin plot for the murine *B. thetaiotamicron* (*Bt*) target.

**

Fig. S7: RNA-guided integration with MetaEdit on the native murine microbiome detected across replicates and time.** Specificity of integration detected by a modified Tn-seq for each mouse replicate (M#1–5) over the entire experimental time course. Both targeted and non-targeted (NT) cages are plotted as metagenome-wide specificity bar plots as quantified by Tn-seq. The hot pink triangle indicates the target site in the native *B. thetaiotaomicron* and the x-axis is the entire metagenome/MAGs in gigabase pairs (Gbp).

**
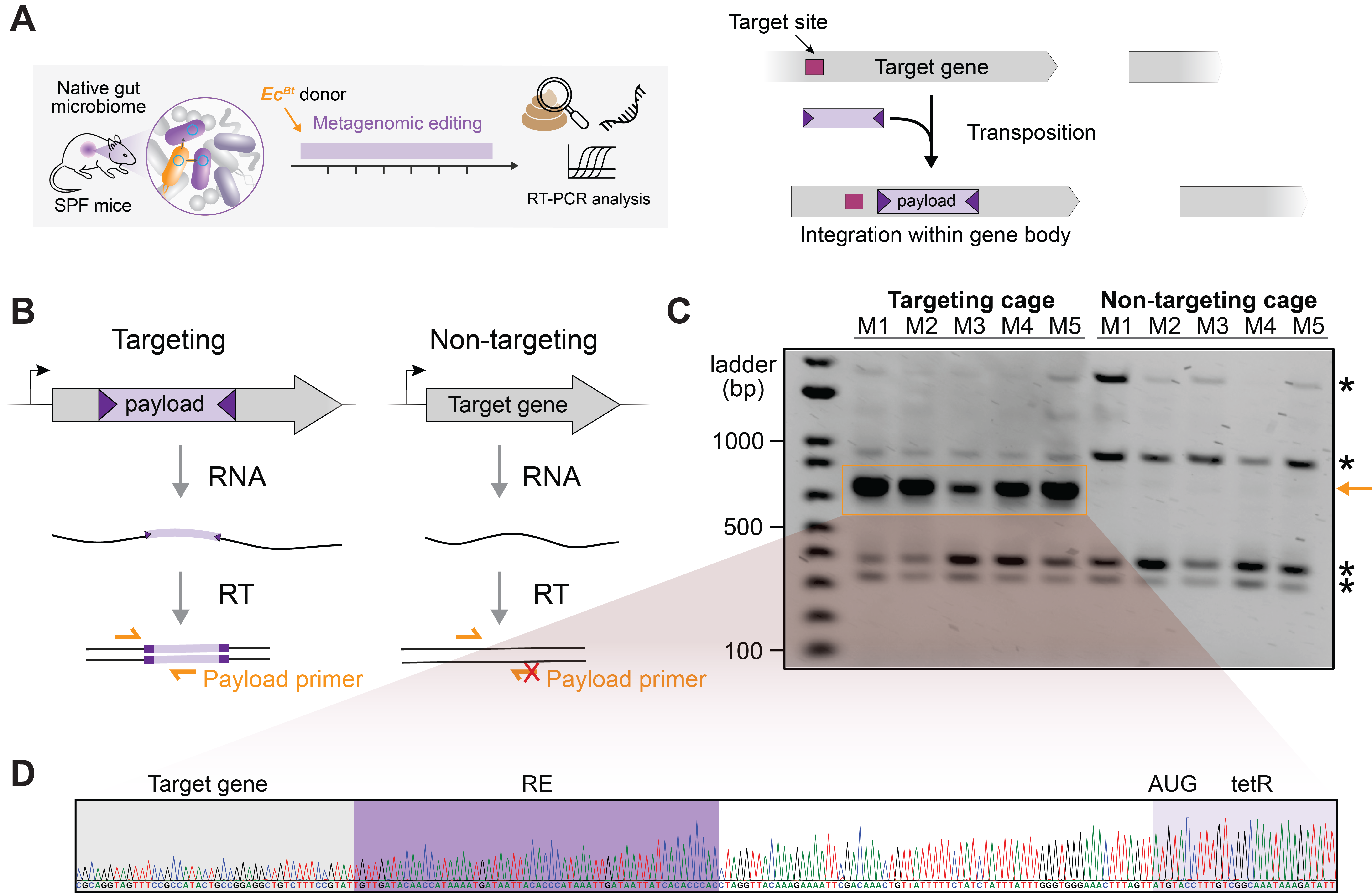
**

**Fig. S8: Expression detection of hybrid transcript from *in vivo* integration in *B. thetaiotaomicron*.** (**A**) Diagram of MetaEdit experimental pipeline and targeting schematic within a gene body. (**B**) Depiction of the RT-PCR scheme for amplifying a hybrid transcript through insertion into a BSH gene in the native target. (**C**) RNA fecal extract RT-PCR agarose gel results of the hybrid transcript in the targeting mouse cohort compared to the non-targeting cohort (each mouse replicate denoted as M#). The orange arrow indicates the expected band length. The asterisk (*) indicates mis-priming PCR products. (**D**) Sanger sequencing confirmation of RT-PCR cDNA product from hybrid RNA. Target gene indicates the portion of the cDNA corresponding to the 5’-end of the BSH transcript, RE indicates the right transposon end of the payload, AUG indicates the start codon of the integrated *tetR* payload followed by the rest of the gene.

**
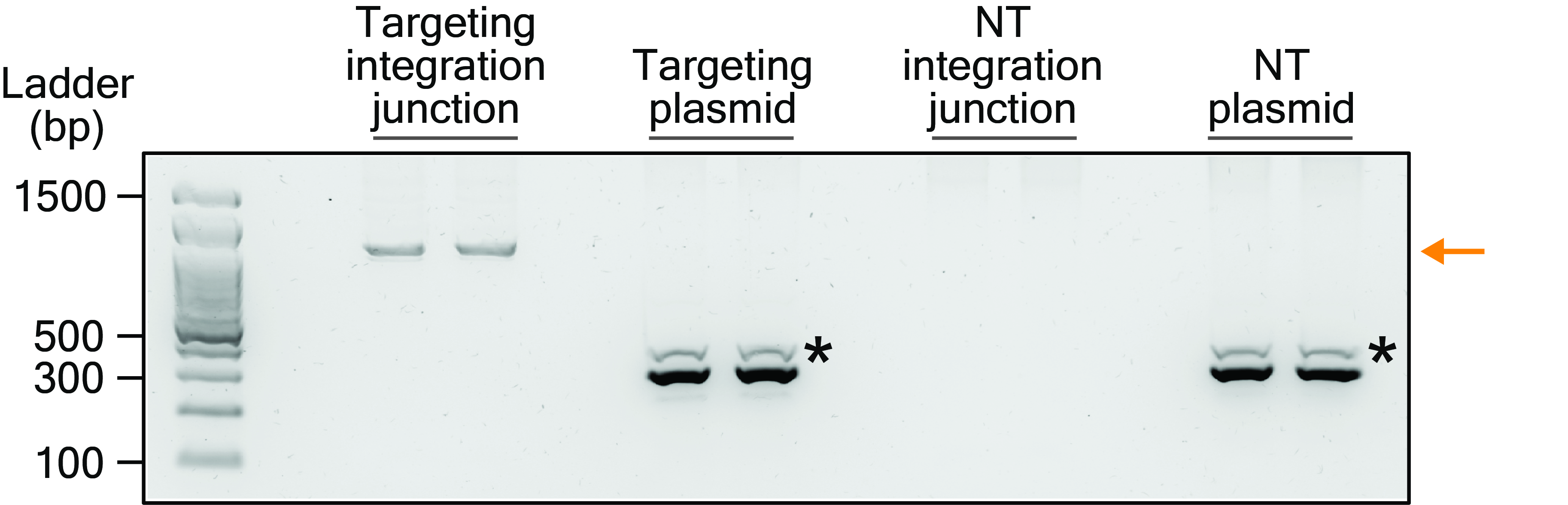
**

**Fig. S9: Genomic integration detection in MetaEdit-tagged *B. thetaiotaomicron* isolates.** PCR agarose gel results of the genomic on-target integration junction in two *Bt* strains isolated from (Fig. 2D) compared to non-targeting isolates. In addition, PCR detection of the MetaEdit plasmid within the isolates. The orange arrow indicates the expected band length. The asterisk (*) indicates mis-priming PCR products.


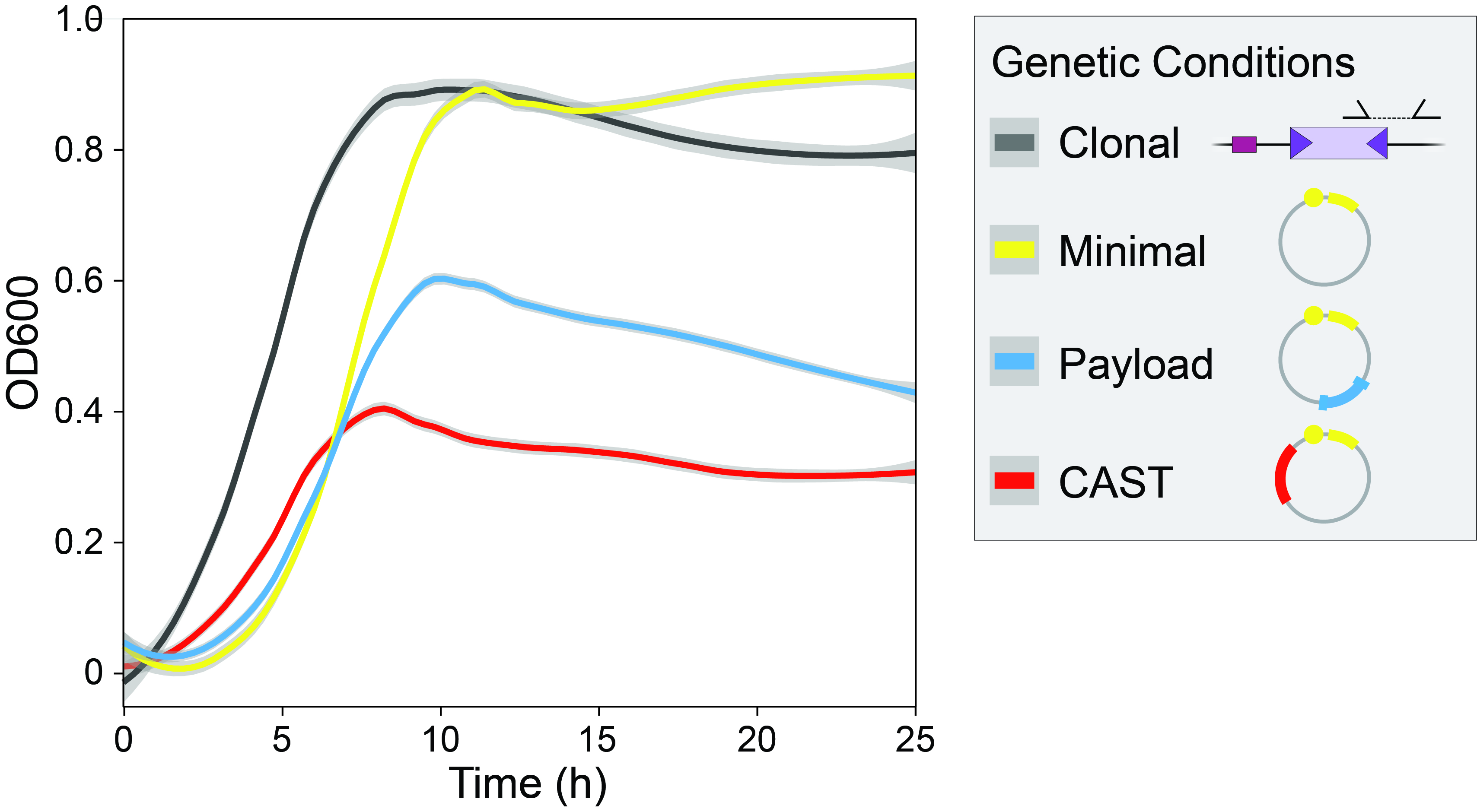


**Fig. S10: Growth profiles of fitness cost of MetaEdit components on engineered *B. thetaiotamicron*.** Growth curve of *Bt* conjugated with the indicated genetic condition: clonally integrated, harboring a minimal plasmid with the oriT and rep ORF, harboring the constitutively expressed payload, or harboring the constitutively expressed CAST operon.


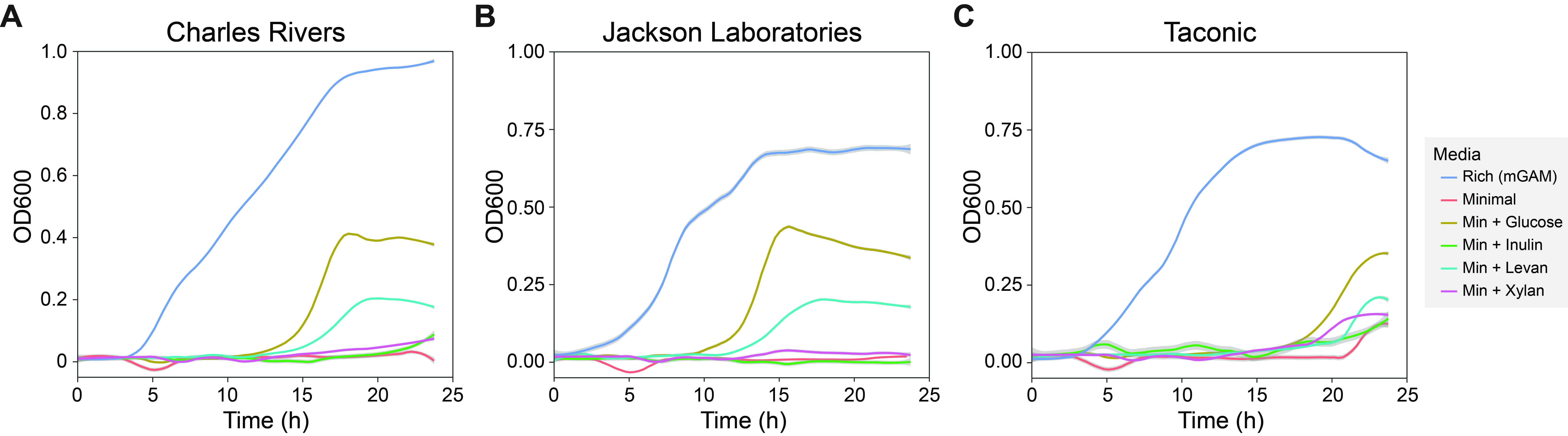


**Fig. S11: Growth profiles of mouse vendor fecal communities with polysaccharide carbon sources.** Growth curves from fecal communities in minimal media supplemented with polysaccharides (Inulin, Levan, and Xylan) for three mice vendors: (**A**) Charles Rivers, (**B**) Jackson Laboratories, and (**C**) Taconic. **‘**Rich (mGAM)’ indicates rich media, ‘Minimal’ indicates that no carbon source was added to the minimal media, and ‘Min + X’ indicates which carbon source was added to the minimal media. Growth curve data in (A-C) are shown as mean ± s.d. as gray shading for n=3 independent biological replicates.

**
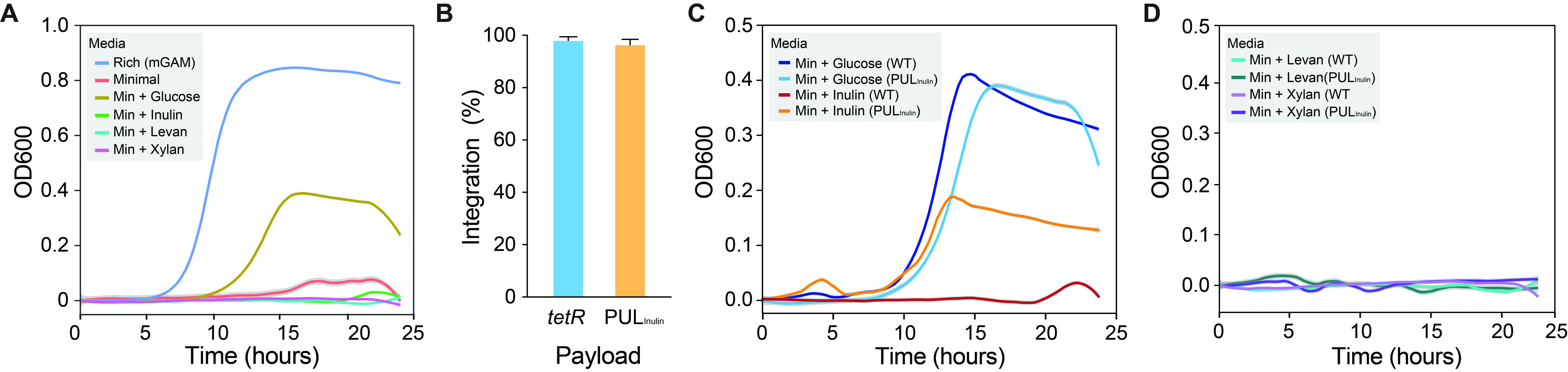
**

**Fig. S12: Growth profiles of native and PUL-engineered *B. thetaiotamicron* (*Bt*) across various polysaccharides.** (**A**) Growth curve of wild-type *Bt* in minimal media supplemented with the indicated polysaccharides. (**B**) Integration efficiency of the 7 kb PUL_inulin_ payload compared to the 1 kb *tetR* payload in *Bt*. (**C**) Growth curve of *Bt*-PUL_inulin_ compared to WT with or without glucose and inulin supplementation. Isolates are clonally integrated and validated before their use in growth experiments. (**D**) Growth curve of *Bt*-PUL_inulin_ in minimal media supplemented with xylan or levan. Growth curve and integration efficiency data in (A-D) are shown as mean ± s.d. as gray shading for n=3 independent biological replicates.

**
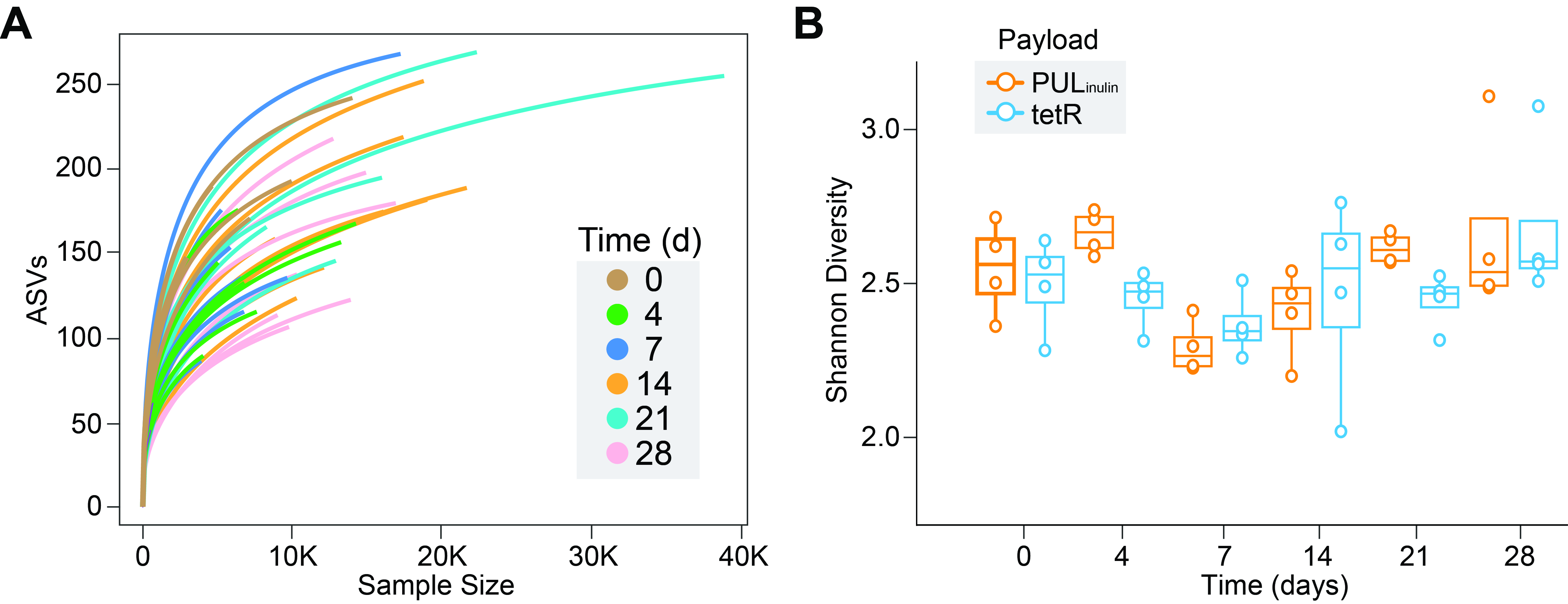
**

**Fig. S13: Native microbiome diversity during inulin supplementation.** 16S profiling of fecal samples from *Bt-*PUL_inulin_ and *Bt*-*tetR* mice supplemented with inulin and diversity metric calculalation for (**A**) Alpha diversity rarefaction plots and (**B**) Shannon diversity for each sample in the native murine over time microbiome. Deep sequencing 16S data are shown as biological replicates for four mice per cohort.

**
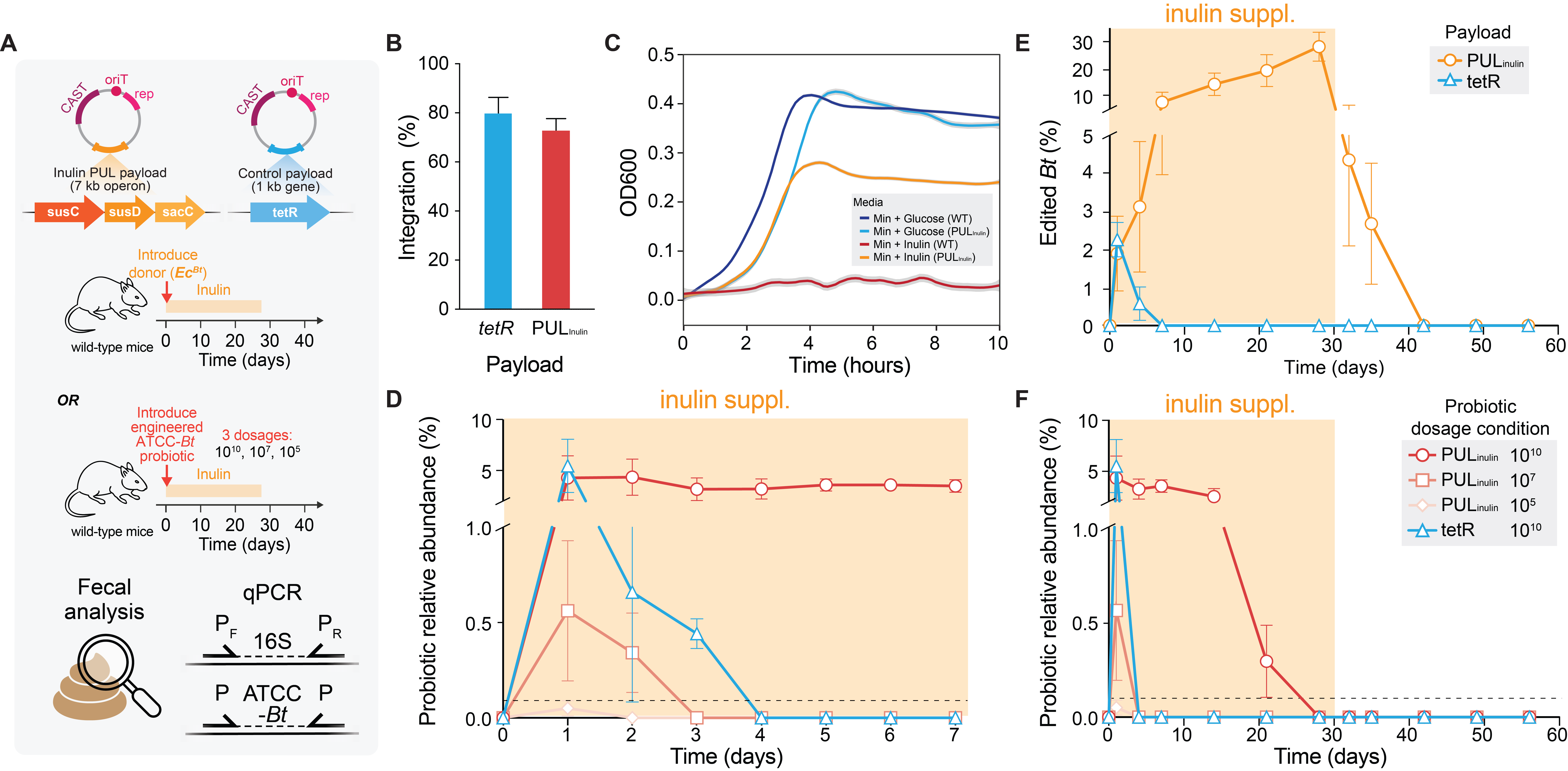
**

**Fig. S14: Comparison of PUL-engineered probiotic ATCC-*Bt* to *in vivo* MetaEdited *Bt*.** (**A**) Experimental diagram of *in vitro* editing ATCC *Bt* to administer as a probiotic or *in vivo* targeting native *Bt* with pME vectors encoding PUL_inulin_ or *tetR* control with dietary perturbations. (**B**) *In vitro* integration efficiency of the 7 kb PUL_inulin_ payload compared to the 1 kb *tetR* payload in ATCC *Bt*. (**C**) Growth curve of ATCC *Bt*-PUL_inulin_ compared to WT with or without glucose and inulin supplementation. Isolates are clonally integrated and validated before their use in growth experiments. (**D**) Persistence of orally administered *in vitro* edited ATCC *Bt* with PUL_inulin_ (reds) payload using three dosages or *tetR* (blue) payload across 7 days. (**E**) Control experiment with enrichment (%) of edited *Bt* relative to all native *Bt* with PUL_inulin_ (orange) or *tetR* (blue) payloads over time using intermittent inulin supplementation, quantified by qPCR on fecal matter. (**F**) The same experiment as (D) across 60 days, quantified by qPCR on fecal matter. Growth curve and integration efficiency data in (B-C) are shown as mean ± s.d. as gray shading for n=3 independent biological replicates. Mouse experiments are shown as mean ± s.d. for n=4 independent mouse replicates in (D-F).

**
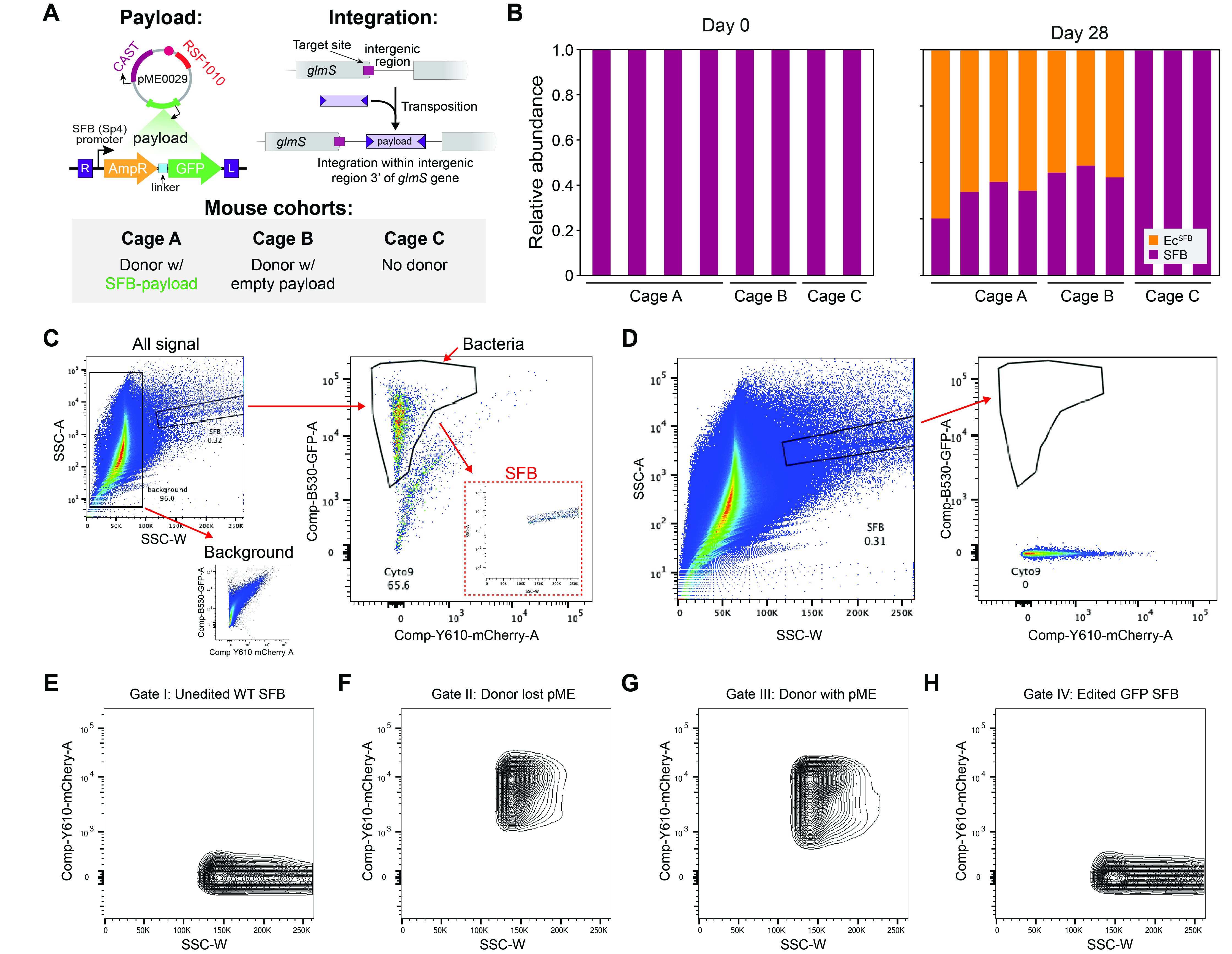
**

**Fig. S15: Expanding MetaEdit into gram-positive Segmented Filamentous Bacteria (SFB).** (**A**) Diagram of the payload genetic architecture with promoters, the targeting schematic within the intergenic site downstream of *glmS*, and the experimental framework for SFB engineering with three cohorts: Cage A, SFB with the donor harboring the SFB MetaEdit vector (targeting condition); Cage B, SFB with the donor without a vector (empty); Cage C, SFB mono-colonized with no donor present (wild-type condition). (**B**) 16S profiling of fecal samples obtained from mice in the indicated cage conditions in (A) before (day 0) and after gavage (day 28). (**C,D**) FACS plots on Cage C fecal samples for parameterization with (C) SYTO 9 DNA stain and (D) without SYTO 9 in order to gate for all bacteria, long SFB filaments, and GFP-positive cells. Black boxes indicate gating, and red arrows indicate double gating. Further analysis on the length of cells (SSC-W) in the four gates (I-IV) determined in (Fig. 4C) targeting conditions. Gates are plotted as contour plots to demonstrate the density of events based on cellular length (x-axis). (**E**) gate I containing WT SFB, (**F**) gate II containing empty donor, (**G**) gate III containing donor with the GFP MetaEdit vector, and (**H**) gate IV containing GFP^+^ SFB.

**
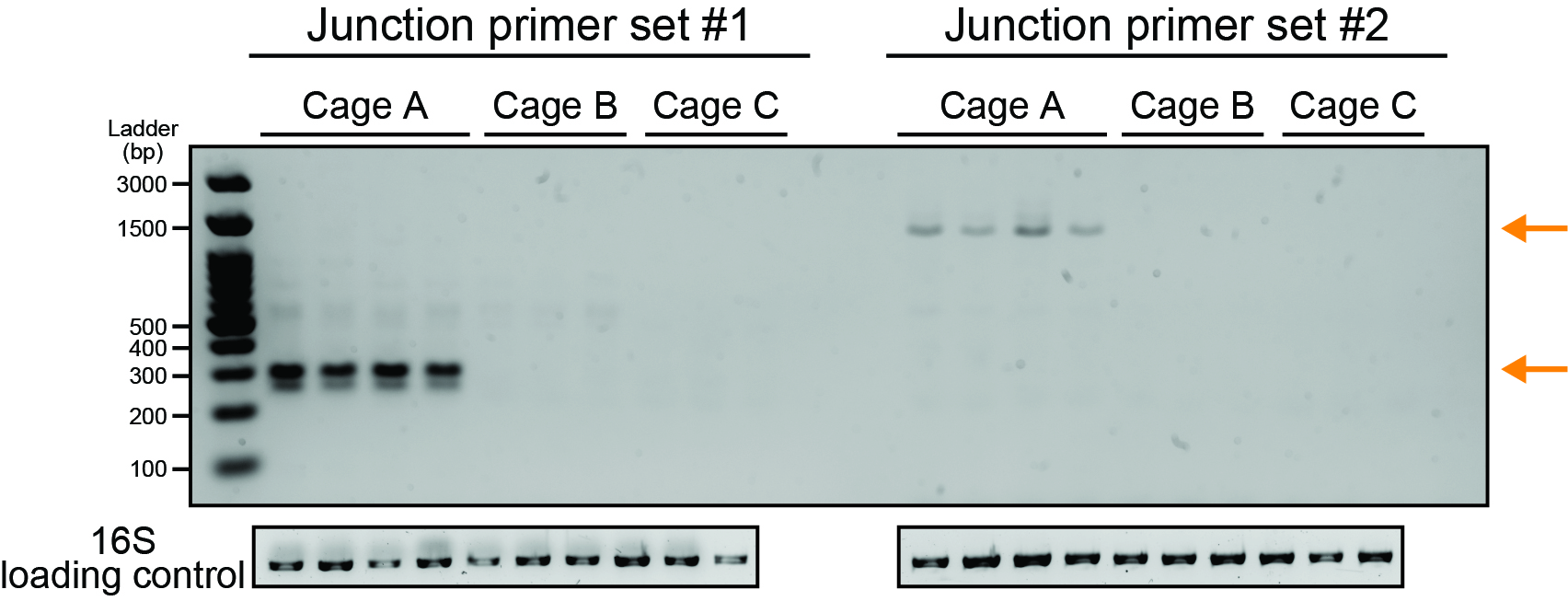
**

**Fig. S16: MetaEdit integration detection in Segmented Filamentous Bacteria (SFB).** PCR confirmation of payload integration in SFB in fecal samples from mice under various conditions. All cage conditions contained germ-free mice mono-colonized with SFB. Cage A indicates SFB treated with a targeting MetaEdit donor, Cage B indicates treatment with a donor with no vector (empty), and Cage C indicates no treatment with donor. Two PCR primer sets were tested to detect the integration junction, with the forward primer annealing to the SFB genome upstream of integration and the reverse primer annealing within the payload. Orange arrow indicates the correct band sizes for each primer set. Universal 16SV4 primer sets were used on the same fecal DNA extracts to control for DNA input and shown as an inset below the top gel.

**Fig. S17: Microscopy on sorted gates.** Representative micrographs of (**A**) sorted payload-integrated SFB compared to the sorted WT. Rows 1-4 are represent SFB detected in separate mice (biological replicates). (**B**) Detection of donor *Ec^SFB^* harboring the MetaEdit vector (mCherry^+^GFP^+^). (**C**) Detection of putative conjugative mating between an engineered SFB with a donor *Ec^SFB^* strain attached to the filament. White arrows indicate the donor strain.


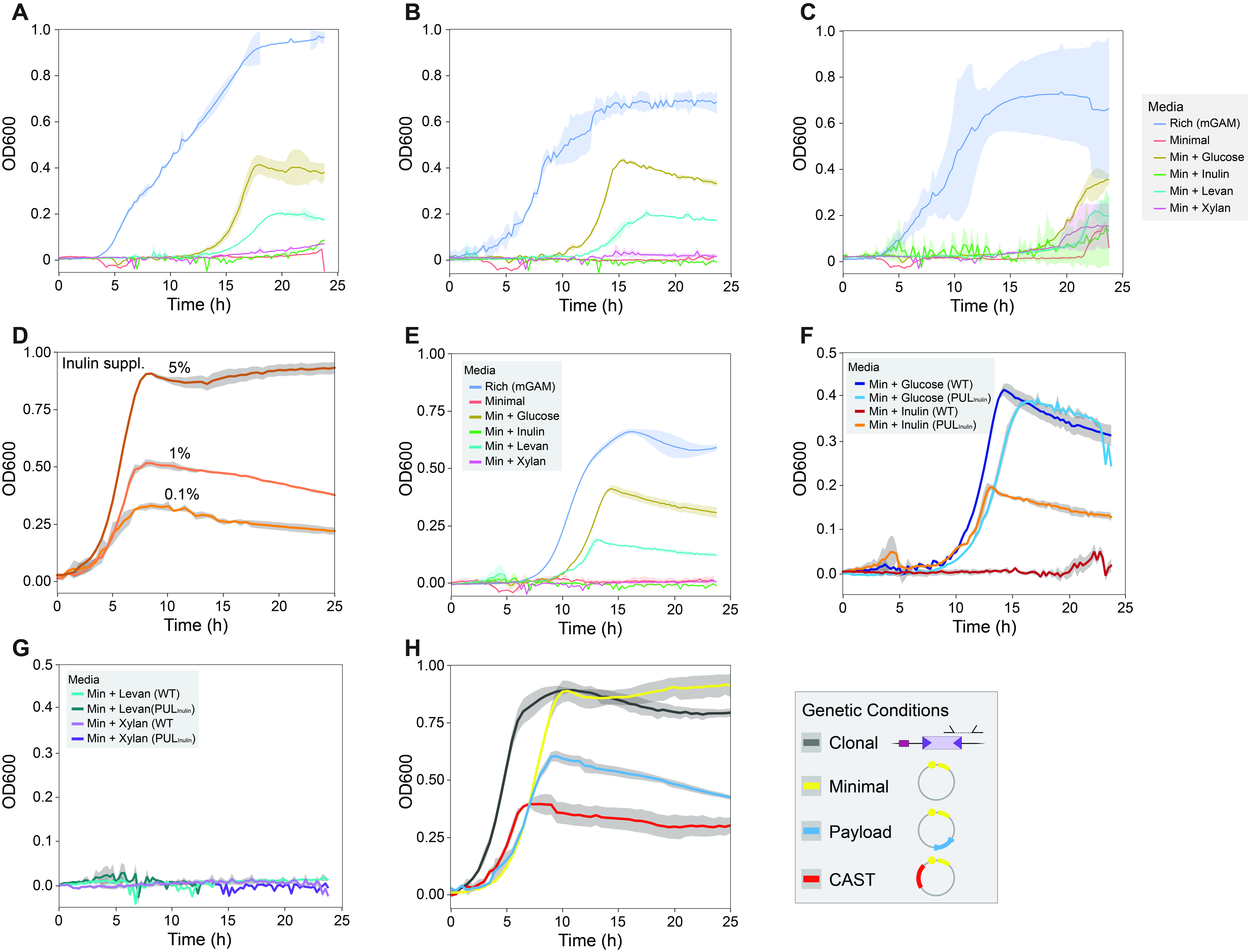


**Fig. S18: Primary data for growth curves.** Growth curves shown as mean ± s.d. for n=3 independent replicates for fecal communities in minimal media supplemented with polysaccharides (Inulin, Levan, and Xylan) for three mice vendors: (**A**) Charles Rivers, (**B**) Jackson Laboratories, and (**C**) Taconic. (**D**) Growth curve of PUL_inulin_-integrated *Bt* in minimal media with increasing inulin concentrations. (**E**) Growth curve of wild-type *Bt* in minimal media supplemented with the indicated polysaccharides. (**F**) Growth curve of *Bt*-PUL_inulin_ compared to WT with or without glucose and inulin supplementation. Isolates are clonally integrated and validated before their use in growth experiments. (**G**) Growth curve of *Bt*-PUL_inulin_ in minimal media supplemented with xylan or levan. (**H**) Growth curve of *Bt* conjugated with the indicated genetic condition: clonally integrated, harboring a minimal plasmid with the oriT and rep ORF, harboring the constitutively expressed payload, or harboring the constitutively expressed CAST operon.
